# Chalkiness and premature controlled by energy homeostasis in *OsNAC02* Ko-mutant during vegetative endosperm development

**DOI:** 10.1186/s12870-024-04845-8

**Published:** 2024-03-18

**Authors:** Mei Yan, Guiai Jiao, Gaoneng Shao, Ying Chen, Maodi Zhu, Lingwei Yang, Lihong Xie, Peisong Hu, Shaoqing Tang

**Affiliations:** State Key Laboratory of Rice Biology, Key Laboratory of Rice Biology and Breeding of Ministry of Agriculture, China National Rice Research Institute, Hangzhou, 311400 China

**Keywords:** NAC, *Oryza sativa*, Premature, Chalky, Cell cycle

## Abstract

**Background:**

Chalkiness is a common phenotype induced by various reasons, such as abiotic stress or the imbalance of starch synthesis and metabolism during the development period. However, the reason mainly for one gene losing its function such as NAC (TFs has a large family in rice) which may cause premature is rarely known to us.

**Results:**

The Ko-*Osnac02* mutant demonstrated an obviously early maturation stage compared to the wild type (WT) with 15 days earlier. The result showed that the mature endosperm of Ko-*Osnac02* mutant exhibited chalkiness, characterized by white-core and white-belly in mature endosperm. As grain filling rate is a crucial factor in determining the yield and quality of rice (*Oryza sativa*, ssp.* japonica*), it's significant that mutant has a lower amylose content (AC) and higher soluble sugar content in the mature endosperm. Interestingly among the top DEGs in the RNA sequencing of N2 (3DAP) and WT seeds revealed that the *OsBAM2* (LOC_Os10g32810) expressed significantly high in N2 mutant, which involved in Maltose up-regulated by the starch degradation. As Prediction of Protein interaction showed in the chalky endosperm formation in N2 seeds (3 DAP), seven genes were expressed at a lower-level which should be verified by a heatmap diagrams based on DEGs of N2 versus WT. The Tubulin genes controlling cell cycle are downregulated together with the MCM family genes MCM4 ( ↓), MCM7 ( ↑), which may cause white-core in the early endosperm development. In conclusion, the developing period drastically decreased in the Ko-*Osnac02* mutants, which might cause the chalkiness in seeds during the early endosperm development.

**Conclusions:**

The gene *OsNAC02* which controls a great genetic co-network for cell cycle regulation in early development, and KO-*Osnac02* mutant shows prematurity and white-core in endosperm**.**

**Supplementary Information:**

The online version contains supplementary material available at 10.1186/s12870-024-04845-8.

## Background

Rice (*Oryza sativa* L.) is one of the most important cereal crops in the world, it serves as the main dish for more than half of the world’s population, and it is a major food for Asians. The chalkiness of rice grains can impact its marketing value for its appearance, eating and milling quality and even the yield. The floury endosperm mutants (*flo1* to *flo5*) have a mechanism of chalkiness which is different from the mutant of TFs deletion. For instance, the *flo2* mutant is caused by BEI down-regulation and decreasing expression of AGPase, GBSS, SS, BEIIb [1], while the *flo4* mutant is caused by the PPDKB deletion(for pyruvate orthophosphate Dikinase B [2]. Plants containing the *flo4-1* produced no PPDKB transcript, resulting in the white-core endosperm phenotype with 6% weight loss. Meanwhile, the new function of proteins FLO6 and FLO7 which are located in the plastid and endosperm periphery respectively, shown a floury endosperm in their Ko-mutants as well. Both of them shown a novel insight into the role of proteins interacted with amyloplast in the endosperm [3, 4].

Premature development of rice grains be induced by various factors, such as ABA-induced genes deletion or premature cell process death. Heat stress-induced hydration is identified as the main reason for the slight chalkiness in the ventral region [5]. Additionally, moderate drought stress has been found to enhance reproductive growth and accelerate the premature senescence process [6]. Premature senescence can also be induced by ER stress, which proceeds in a precisely orchestrated developmental program and causes the chalky endosperm [7].

The filling rate is an essential factor for determination of yield and seed development period based on previous studies [8, 9]. In this study, the grain filling rate was accelerated in the Ko-*Osnac02* mutant compared with the wild type (WT), which caused the chalkiness in the endosperm of mature seeds (125 DAG, days after germination). This induced the early maturity which not only affected the yield, but also decreased the quality simultaneously. Thus, RNA-seq analysis of the Ko-mutant seeds (3DAP, days after pollination) was primarily performed and online bioinformatics were used for the motif detection in promoter regions. Meanwhile, the multi-regulation of *OsNAC02/OsNAC06* at the mRNA-protein level was revealed by multi-omics such as Prediction of Protein interaction (PPI) and Mapman framework. The environmental stress response at postal transcript level could continuously display the mechanisms of the premature and chalky phenotype, in the Sucrose and Starch metabolic pathway. 

*OsNAC02* (LOC_Os02g38130, Os02g0594800) is involved in the regulation of cell cycle control. It is located in the nucleus where it regulates the development of various organs including seeds, flowers, SAM and seedling roots. The vegetative and reproductive development processes in Ko-*Osnac02* mutant may suppress starch synthesis by the Starch and Sucrose pathways, which are implicated to change the endosperm development as a result. In rice except *OsNAC02*, another gene highly homologous to *AtSOG1* is *OsNAC06*(Os06g0267500) [10]. The function of these two genes just like one coin have A and B sides. 

OsNAC02 can regulated more than five effective TFs, which has been confirmed by H-1-Y, including HSF, NAC and MYB factors. This suggests that the abiotic resistance of the Ko-*Osnac02* mutant changes according to the pathway activated by these TFs. In a previous study, except HSF, the TFs including NAC, AP2/ERF, WRKY, MYB, bHLH, MADS and C2H2 predominantly responded to heat stress [11–13]. All of these TFs are directly regulated by OsNAC02 and have the ABRE/G-box in their promoters, which means that they could not only respond to heat shock, but they could also be induced by other abiotic stress. NAC(NAM-ATAF-CUC) was a plant-specific transcription factor which has a large family and is distributed widely in plants (Fig S3), but it has a similar function in homologous plants [14, 15]. The rice NAC-domain proteins are highly conserved in different plants, and they all have similar abiotic stress responses and tolerance to harsh environments [5, 8, 16–22]. 

In this study, the Ko-*Osnac02* mutants are constructed on the Nipponbare background. When compared with the WT, the Ko-*Osnac02* mutant displayed a disrupted homeostasis of starch metabolism, resulting in premature and chalky seed phenotypes. 

## Results

### K0-*Osnac02* /*Osnac06* mutant construction and the genotype detection

In this study, the functions of *OsNAC02* (Os02g0594800, LOC_Os02g38130) and *OsNAC06* (Os06g0267500) were mainly identified by Ko-mutants construction. However, functional detection was performed through the online bioinformatics analysis and RNA-Seq analysis in mutants, which provided ways to discover the functions and revealed various phenotypes in response to abiotic resistance as well as the prematurity which may seriously reduce the quality of seeds.

The multi-mutant of N2 *mut* (*Osnac02*) and N3 *mut* (*Osnac02/Osnac06*) was created by CRISPR/Cas 9. All of the mutant sites were confirmed through PCR sequencing, and the leaves of N2/N3 *mut* (T2) were selected for the mutant detection in detail. The N2 *mut* has two mutations in the CDS region of *OsNAC02*, while the N3 *mut* has one mutant sites in the CDS regions of *OsNAC02* and *OsNAC06* respectively (Fig S1a). 

**Fig.1:**
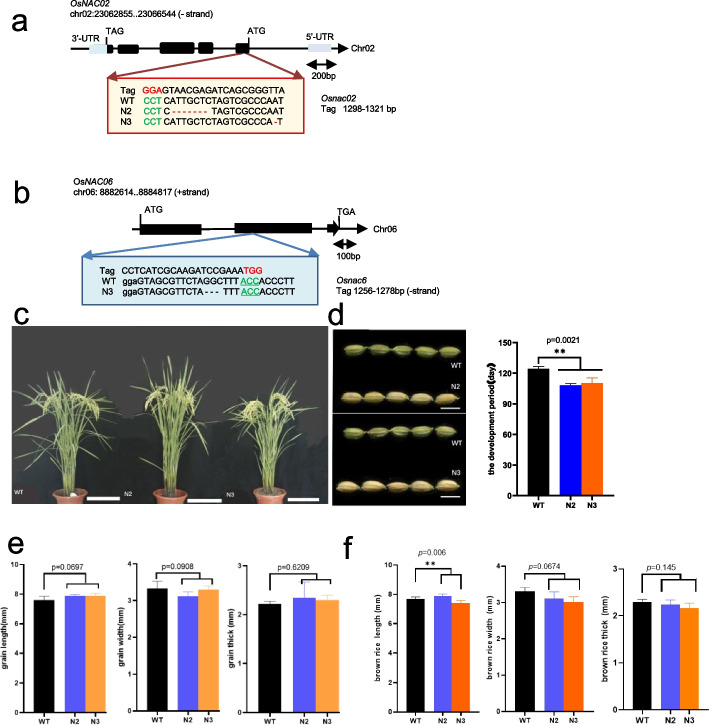
The K0-OsNAC02/OsNAC06 mutant (T2) construction and botanic characters detection. Two mutants named N2, N3 are planted in field (Fuyang, Hangzhou in 2021), and the homozygous was harvested in the autumn. The control is wild type of Nipponbare. The genetic detection of all homozygous mutants was performed by PCR sequencing. The N2 has two mutant sites in *OsNAC02* CDS regions, N3 has one mutant site in *OsNAC02* and *OsNAC06* CDS regions respectively (Fig. S1). **a** N2 mutant construction. The K0-*Osnac02* mutant created by *Osnac02* -tag2 has 7 bp-deletion in N2 *mut* and K0-*Osnac02* mutant has 1bp-deletion in N3 *mut*, respectively.** b** N3 mutant construction. The K0-*Osnac06* mutant created by *Osnac06* -tag2 has 3 bp-deletion in N3 *mut*. **c** The N2/N3 *mut* plants (95DAG) are selected for botanic characters detection (Fig. S2). *n* = 17.5 cm. **d** The premature phenotype in N2/N3 *mut* (95DAG) and the development period calculation of the mutants (two-way ANOVA for mixed model, *, *p* < 0.05 and **, *p* < 0.01, *n* = 6 mm). The mutant matured earlier than WT as a result. For the detection of premature phenotypes in N2 and N3 *mut*, the seed shape characters such as length, width, and thickness are collected in Fig e and Fig f. **e** The shape phenotypes detection of N2/N3 mut seeds (95 DAG), and there are no difference between mut vs WT. **f** The shape phenotypes of full-mature seeds (T2) in N2/N3 *mut* (125 DAG) are detected, which were harvested in 2021. The full-filled seeds (125 DAG) of WT is larger than mutants on the whole

Prematurity happened in both N2 and N3 *mut* seeds (95 DAG). In the summer of 2021, N2/N3 *mut* seeds (T2) were harvested (95 DAG), and the panicles matured early obviously. The period statistics of endosperm development showed that the N2/N3 mutants matured 10 -15 days earlier than the WT (Fig. 1d). At 95 DAG, the caryopsis phenotype in the last stage of the grain-filling period differentiated substantially more than the WT. We found that the WT caryopsis was still green while all of the mutant caryopses were in a yellowish state.

Meanwhile, the shape phenotypes of seeds at 95 DAG and 125 DAG are calculated separately for the maturity verification. The seeds (95 DAG) of N2/N3 *mut* have superior length and thickness of caryopsis than WT (Fig 1e), but become incompletely filled in the mature seeds (125 DAG) (Fig 1f). And all the botanic morphology and yield characters of N2/N3 *mut* (T2) are detected in 2021 (Fig 1c and Fig S2). 

### *OsNAC02* (Os02g0594800) expression pattern and phylogenetic analysis of OsNAC family in rice

**Fig. 2:**
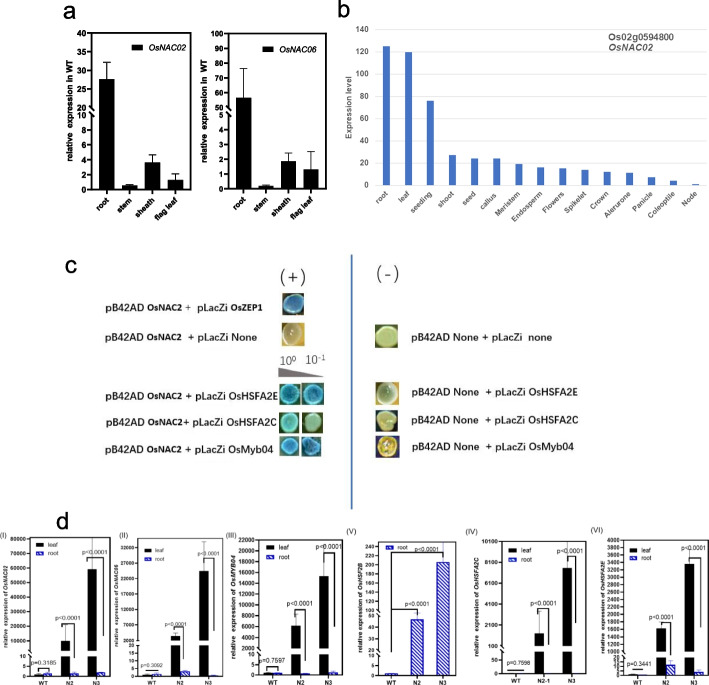
The functional analysis of *OsNAC02* and its co-network construction. **a** The distinct expressions of *OsNAC02* and *OsNAC06* were verified by qRT-PCR in different tissues. Both of them expressed highly in root, as this organ is most vegetative parts of WT seedling. The ubiquitin (LOC_Os03g13170.1, Os03g0234200) was used as the reference which expressed in the whole plant. **b** The distinct tissue expressions of *OsNAC02* (LOC_Os02g38130, Os02g0594800) on NCBI, and this result is in accordance with Fig a. **c** Yeast-one-hybrid assays show the *OsNAC02* interact with the promoter fragments of *OsHSFA2C, OsHSFA2E, OsMYB04*. The positive control in blue colonies was constructed according to the reference [24]. There are two cultures in this experiment used as the selection medium: SD and SD /-Ura/-Trp. The X-gal culture is SD /-Ura/-Trp + DMF + X-gal with the content of 10–1 and 10-2 for positive screen. **d** The qRT-PCR results represent the expressions of OsNAC02 regulated genes in the leaf and root of mutants vs WT, including: *OsNAC02*, *OsNAC06*, *OsMYB04*, *OsHSF2B*, *OsHSFA2C*, *OsHSFA2E* (Table S12). The ubiquitin (LOC_Os03g13170.1) is used as the reference gene (student *t-test*, *, *p* < 0.05 and **, *p* < 0.01), and the motif locations are listed in the results (Fig. S5a)

In this study, qRT-PCR was the main method for investigating the relative expression of *OsNAC02* in all tissues of the seedling plant, including the root, stem, sheath and flag leaf (45 DAG). As in the Fig 2a shows, the relative expression level of *Osnac02 mut* and *Osnac06 mut* based on qRT-PCR analysis were in accordance with the results online (Fig 2b) [23], both were overexpressed in the root, SAM and the panicles during the early stage of development.

There are a total of 195 genes containing the NAC domain in rice, and 131 genes were selected for NAM phylogenetic analysis, and these are as same as the analysis in 2003 [14] (Fig S3). There are 131 OsNAC genes are clustered into four subgroups. *OsNAC02* (Os02g0594800) belongs to the Subgroup II including only seven genes, and *OsNAC06* (Os06g0267500) was clustered into Subgroup III. The *OsNAC02* classified into a higher place in the phylogenetic tree, which means its more unique and conserved during the plant evolution.

The homologous genetics of *OsNAC02* and *OsNAC06* were highly conserved (Figs S3 and S4), and these genes were conserved in almost all of the plants, then *OsNAC02* were identified through homologous analysis with orthologous genes from rice, maize and Arabidopsis. *OsNAC02* (LOC_Os02g38130) and *OsNAC06* (Os06g0267500) were aligned with other seven proteins: Maize-NAC, NAC1, NAC2, ANAC044, ARATH-NAC085, NAC_044 and At1g26390, and the clustering results indicated that the N-terminus of the protein domains (0-244 aa) was highly conserved, but the sequences changed variously on the C-terminus (Fig S4a). The location of the motifs in the promoter of the relative genes are primarily homologous in rice. 

The OsNAC02 regulated genes including *OsHSFA2C*, *OsHSFA2E* and especially *OsMYB04* in the PAL pathway, are highly conserved in all plants (Fig S5). which were found to be directly regulated by *OsNAC02*. These downstream transcription factors are multiple effector responses to both abiotic stress and other hormonal regulation. All of these genes containing "ACGT" motif, such as the G-boxes and the ABRE motifs in the promoter region, and their transcriptional functions were determined by the multiple effective motifs and regulatory genes. Among the many cis-elements, the ABRE/G-box motifs located in the promoter of *OsNAC02* effective genes were collected and listed (Fig S5a). 

Meanwhile, the regulation of OsNAC02 with relative TF genes can be dedicated by comparison of qRT-PCR results in root and leave of seedlings in mutants (45 DAG). As Fig 2d shows, the expression of all the TFs in low-level was consistent with OsNAC02 losing function in root, but normally expressed in leaves which is accordance with OsNAC expressing lower with lower affection in root. Meanwhile, the overexpression of *OsHSFA2C* and *OsHSFA2E*-related *OsMYB04* is found in all of the mutant leaf compared with WT. As Fig 2c shows, all the genes are positively regulated by the OsNAC02 directly, and their expression levels are similar to the level of *OsNAC02* expression in different tissues. But in roots, the expression of *OsHSB2c* used as a control is higher than other genes, the reason especially for which may be regulated by OsNAC02 indirectly. 

### Chalky phenotype and quality characters of brown rice in mutants

The grain chalkiness was detected in three repeated samples selected randomly including the percentage of chalky grain, percentage of chalky area, besides the rice broken ratio (Fig S6a). The rice chalky ratio of N2 *mut* was mostly significantly high, and the yield of N2 *mut* was lower than that of the WT (Fig S2c). Moreover, the yield of N3 *mut* was higher than that of WT, while the rice broken ratio of the N3 *mut* double mutants was highest among all materials (Fig S6a). 

**Fig. 3:**
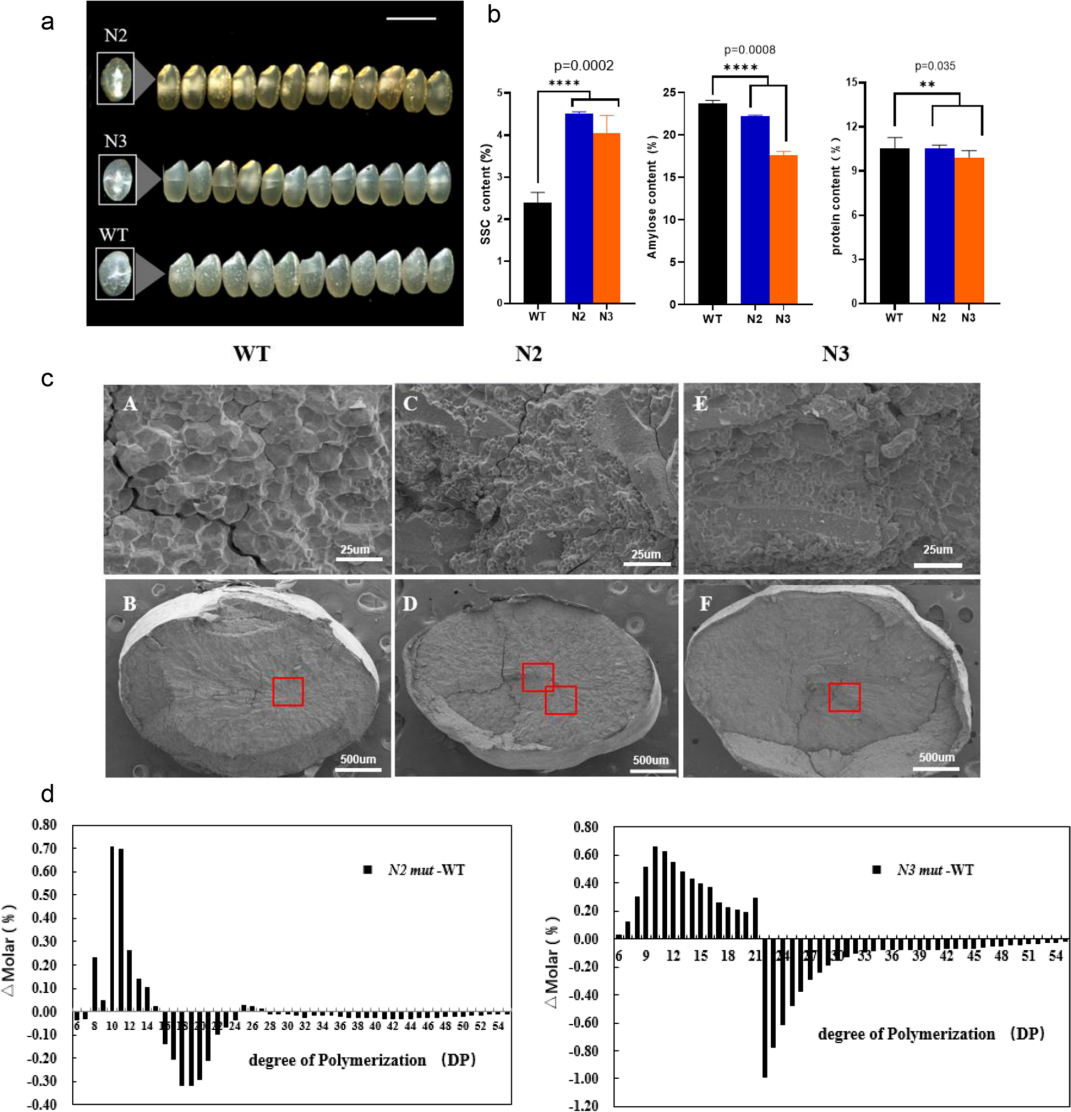
The chalky phenotype and physiochemical analysis in the N2/N3 mutants vs WT. All the characters in this figure are detected in full mature seeds (125 DAGs). **a** The chalky phenotypes and shape phenotypes of N2/N3 *mut* compared with WT. All the T2 mutants are planted in Fuyang (Hangzhou, 2021), *n* = 15 mm. There are obvious white-core and white-belly in N2 mutant which chalky area is the largest, while there is medium transparent in WT and white-core only in N3 mutant. **b** The physiochemical analysis of N2/N3 mutants is distinct compared with WT. The quality characters such as SSC, amylose content, protein content were tested and the results were as follows: The SSC was highest in N2 mutant but lowest in WT; The amylose content was lowerin N2/N3 mutants compared with WT, which was reversed to the SSC tendency in mutants; The protein content was lowest in N3 but no significant difference between WT and N2 (two-way ANOVA for mixed model, *, *p* < 0.05 and **, *p* < 0.01). **c** The hand-cut sections of full-mature endosperms are scanning by SEM (scale bar, 25 mm and 500 um). The starch granules arranged distinctly between N2 and N3 *mut* compared with WT, and the structure of starch granules are smaller and more polyhedral in mutants. **d** The amylopectin analysis shows the apparent difference in N2/N3 *mut* and WT. The chain length distribution of amylopectin analysis was calculated by the number of short chains with degree of polymerization (DP). There are more short fragments in N2 *mut* while more medium fragments in N3 *mut*

As shown in the Fig S6b, the milled rice of N2 *mut* has a floury endosperm with white-core and white-belly appearance, and the chalky phenotype was more obvious than N3 *mut* (Fig 3a). Moreover, the chalky phenotype of the N3 *mut* milled rice was similar to that of WT in 2021. Both N3 *mut* and WT showed a small white-core chalky phenotype at the center of the endosperm which was caused by the persistent high temperature in summer of 2021, but the decrease in chalky rate may not rescue the quality of N3 *mut*, for the significantly fragile phenotype with more than an 85% broken ratio in N3 *mut* milled rice (Fig S6c). Overall, the chalky area and ratio of N2 mut were significantly higher, the rice broken rate of N3 mut was mostly increased, and the phenotypes analysis of mature seed (125 DAG) such as length, width, and thickness of grain was in the tendency of WT > N2 > N3 (Fig 1f). Moreover, the yield per plant of T1 mutants (125 DAG) was in the order of N3 > WT > N2 in 2020 (Fig S2c), but N2 > N3 > WT at the 95 DAG of T2 mutant in 2021 (Figs S2e and S2f).

**Fig. 4:**
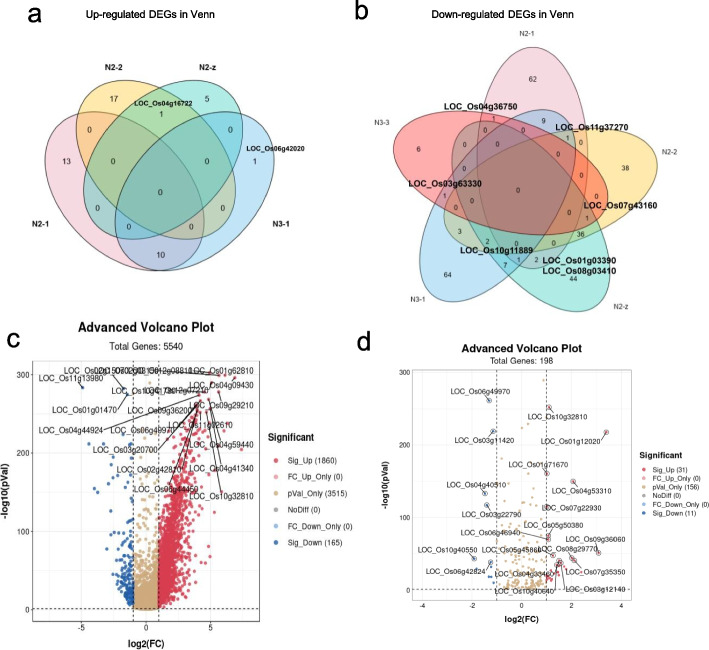
The DEGs clusters and GO enrichment of RNA-Seq in mutant vs WT seeds (3 DAP) Down-regulated DEGs in Venn. **a** The Venn diagram of up–regulated DEGs are based on the RNA-Seq of seeds (3 DAP). **b** The Venn diagram of down–regulated DEGs based on the RNA-Seq of seeds (3 DAP). All the genes with label are listed in Table S5. In total, five mutants N2-1, N2-2,N2-z, N3-1,N3-3 were used for the cluster analysis, and the N2-2 and N3-1 duplicated samples are N2-z and N3-3. **c** The volcano cluster of the DEGs in RNA-Seq of seeds (3 DAPs). As cluster shown, most of the DEGs in red are up-regulated genes, the down-regulated genes are in blue and the genes expression with no difference are in yellow. All the genes expressed significantly are listed in Table S8. **d** The volcano cluster of the DEGs in Sucrose and Starch pathway of RNA-Seq in N2/N3 *mut* vs WT seeds (3DAPs). All significantly expressed genes are listed in Table S9

The hand-cut sections of the matured endosperm (125 DAG) were obtained by scanning electron microscope (SEM) (Fig 3), the results showed that the starch granules were arranged distinctly in the mutants compared to that in WT. The number of starch granules was greater in N2/N3 *mut*, which developed into a floury endosperm. The volume of starch granules was smaller in N2 *mut*, and several narrow fissures were observed among the parenchyma cell. However, the N3 *mut* granules have a faster filling rate than that in N2 *mut*, leading to a tighter arrangement of the cell wall with nearly no crack in the endosperm. However, the starch granules were round, smooth which is different from that in WT (Fig 3c). As in the normal control, the parenchyma cells of the WT were almost flat, and the starch granules were compact with every cell wall having sharp edges (Fig 4c).

Though endosperm sections at 3-10 DAPs were scanned in the slice observation, only 3 DAPs, 5 DAPs section showed a little difference (Fig S7). The iodine-stained semi-thin sections of the opaque endosperm in LM (5 DAP, light microscope) showed that amylose content (AC) was higher in N3 *mut* than in WT versus N2 *mut*, which could be verified by the darker blue color in the early endosperm. Furthermore, as shown in Fig S7 for the opaque endosperm scanned by the TEM, N3 *mut* showed more compact intercellular locking of parenchyma cells than N2 *mut*, and the parenchyma cell volume was larger and even overextend the space between the cells (Fig S7). 

### Physiochemical properties of milled rice in mutant versus WT for quality characters verification

The chalky phenotype of the mutant seeds was detected after ripening. In 2021 summer, all of the materials were planted in the field (Fuyang), and there was a higher temperature during the grain-filling stage, then the brown rice had chalky and the fragile phenotype of mutants milled grain was obvious. For the significant distinction with of the WT, a serious of indexes for physiochemical analysis were detected in our lab, including: the soluble sugar content (SSC), amylose content (AC), protein content (PC), and amylopectin chain length distribution analysis – the degree of polymerization (DP) (Fig 3). 

The SSC was significantly higher in N2/N3 *mut* than in the WT, while the AC decreased significantly in N2/N3 *mut*, however, the soluble protein content hardly changed (Fig 3b). As shown in Fig 3d, according to the length distribution of amylopectin analysis, the DP in N2 *mut* showed that the number of 8-14 small fragments (DP) was much more in the mutant than that in the WT, while the number of ultra-short chains with 6-7 DP and the number of medium and long chains with 16 -24 DP significantly decreased simultaneously. However, the number of short chains with 6-21 DP in N3 *mut* was more than that in WT, while the number of long chains with ≥ 22 DP was significantly lower than that in the control. This trend is different from that in the N2 single mutant (N2-1 *mut*). As shown in all of the results below (Fig 3d), according to the different chain length distributions of endosperms, the N2 and N3 mutants have obvious differences in the chalky phenotypes and starch particle size (Fig 3c). 

### RNA-Seq analysis of seed (3 DAP) in 2021

The Raw data was collected from illumine Novaseq TM 6000 sequence platform, which was used to construct the transcriptome from RNA libraries, the clean data can be further filtered using Cutadapt software. The mapped reads of each sample were assembled using StringTie (http://ccb.jhu.edu/software/stringtie/, version:stringtie-1.3.4d) with default parameters [25]. Then, the transcriptomes from all samples were merged using gffcopare.shtml to reconstruct a comprehensive transcriptome. There are 34280 genes in this transcript files, which were used to estimate the expression levels and abundance of all transcripts. The expression levels were calculated using String and ballgrown which calculate the FPKM value (http:www.biocondutor.org/packages/release/ bioc/html/ballgown.html). 

For this RNA-Seq study, we selected seeds (3DAP) in vegetative phase to investigate seed development. The Fig S9 shown that N2-1 and N2-2 have 6492 and 7048 up-regulated genes, 2260 and 1699 down-regulated genes, respectively. Meanwhile, in N3-1 and N3-2, there are 3797 and 4021 up-regulated genes, 771 and 987 down-regulated genes, respectively. This result indicates the similarity between N2 and N3 replicate samples. The QC values of RNA-seq raw data in different mutant are higher than 96.6%, and all of the GC content are more than 51%.

Based on the results of the RNA-Seq analysis in Fig S9, the expression level of most genes was in the order of N3 > WT > N2. The top 25 genes with up expression-levels in the N2 mutants (red region) seeds are mainly related to pathogen invasion resistance, protein in the root tip for heavy metal ion resistance, and salt stress response, these genes include the terpene biosynthesis enzyme (LOC_Os04g27670), glycosyl reductase (LOC_Os10g28120), and Anth/Enth protein (LOC_Os02g07900), which are mainly involved in endocytosis mediated by the AP2-independent Clathrin protein. All of the functional proteins involved in biological disease resistance were downregulated. Meanwhile, the proteins related to plant hormones such as regulation auxin induced proteins and BRI receptors were downregulated (Table S2). Such as laccase synthesis precursor (*lac15*, LOC_Os10g20610). Previous studies indicated that the PAL pathway was one of the most crucial synthesis pathways for tea (*Camellia sinensis* L.) to respond to gray mold disease stress, and LAC15 (TT10) was the primary enzyme for anthocyanin synthesis [26, 27]. 

On the contrary, the expression level of top 25 genes (blue region) was lower in N2 mutant than that in WT, and most of them were related to the starch metabolism just as follows: the seed gluten synthases (LOC_Os10g26060 and LOC_Os01g55690), enzymes (5×) related to the starch synthesis pathway including the sucrose synthase (LOC_Os07g42490), *Waxy* (LOC_Os06g04200), glucan phosphate acyltransferase (LOC_Os08g25734), OsBE2b (LOC_Os02g32660); the TFs such as *dof* Zinc finger (LOC_Os02g15350), *OsNAC023* (LOC_Os02g12310) and *OsNAC025*(LOC_Os11g31330); the intracellular mitochondrial transporters related to energy conversion (LOC_Os05g02060), pyruvate kinase (LOC_Os05g33570), AGPL (LOC_Os08g25734) and etc. (Fig S9-a and Table S2). 

The heatmap analysis of the N3 versus WT showed a great difference between N3-1and N3-2 *mut*. The expression level of top 25 genes upregulated in the N3-2 *mut* (Fig S9-b, red region), the genes and functions in details are shown in Table S3. In previous research, all the other genes were expressed at low-level while the heat shock protein chaperone HSF7.1 (LOC_Os03g16860) was expressed stably under heat stress. In this study, HSF7.1 expression increased in seeds of N3 *mut* under normal conditions, which was not in accordance with the previous study results, and it may indicate that the deletion of *OsNAC02 /OsNAC06* may remove the inhibited factor from the HSF7.1 [28, 29]. 

Although the phenotype of the broken rice is commonly found in rice harvests, there are still insufficient theoretical studies on these reasons. According to the heatmap of N3 versus WT, we revealed that the DEG expression in N3 mut was significantly lower than that in WT (Fig S9-b, blue region), mainly including: *ZmEBE-1* (LOC_Os01g26320), *GDSL-like* synthase (LOC_Os01g46220) and *CSLA9* (cellulose synthase 9, LOC_Os06g42020) in cytosols. It was confirmed that the three genes involved in endosperm formation in N3 *mut*, which are related to cell wall synthesis and endosperm filling (Tables S3 and S5). The expression of all the genes involved in cell wall synthesis was downregulated, causing the fragile phenotype with significantly higher broken ratio in N3 *mut*. 

Based on the RNA-Seq seed results, the significant DEGs of mutant and the WT were selected, and the 75% of them were related to the starch metabolism, and the rest were related to the monosaccharide and energy metabolism pathways. 90% of these genes were up-regulated, which were related to the starch metabolism pathway in the leaves and roots of mutant seedlings. The expression levels of genes in the N2/N3 mutant were at low-level, and they were even lower than those in the WT. 

### Gene ontology (GO) and KEGG pathway enrichments in the seed (3DAP)

**Fig. 5:**
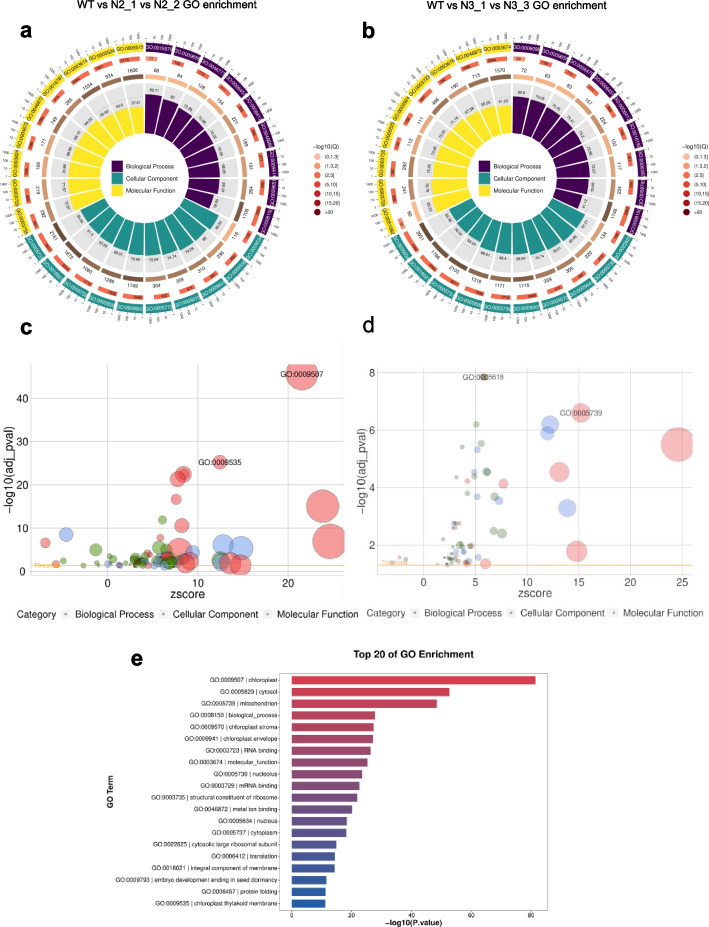
The GO enrichment of DEGs in N2/ N3 mutant vs WT. The gene enrichments of the GO analysis including the top 9 GO pathways of the biological process (purple), the cellular component (green), the molecular function (yellow). **a** The top 9 pathways of GO enrichment in N2 vs WT. **b** The top 9 pathways of GO enrichment in N3 vs WT. **c** The GO bubble Statistics includes biological process (blue), cellular component (red), molecular function (green), which were in accordance with results in fig a and fig e. In the cellular component (red), the two GO pathways: GO:0009507-the chloroplast pathway and GO:0009535-the chloroplast thylakoid membrane pathway were significantly enriched in N2 vs WT. **d** The bubble analysis of Go pathways in the N3 vs WT. In molecular function (green), the GO:0005618-cell wall pathway is the most significantly enriched pathway. While less genes are enriched in the other cellular component (red) GO:0005739-mitochondrion pathway. **e** The results represents the top 20 GO pathways of seed transcriptome including the GO significantly enriched pathways as appearing in fig c and fig d

As shown in the Gene ontology (GO) analysis of RNA-seq in Fig 5 and Fig S9, among all of the GO enrichment pathways in the N2 versus WT seeds (3 DAP, 2021), most of the DEGs in the KEGG pathways were significantly enriched in the chloroplast and chloroplast thylakoid, which is in accordance with the results in Fig 5c. In Fig S9d compared to those in N2 versus WT, the GO enrichment pathways in the N3 vs WT showed that more distinctly expressed genes in the GO pathways were significantly enriched in the chloroplast and mitochondrion, which is in accordance with the results in Fig 5e. Meanwhile in Fig S9c and Fig S9d, many other genes with less rich factors (R < 0.06), which were still enriched in the cytoplasm and nucleus pathways.

In Fig 5c, the GO bubble statistic of the biological process (blue bubble), cellular component (red bubble), and molecular functions (green bubble) are in accordance with the results in Fig 5a and e. Thus, all of the GO enrichments in Fig 5c for N2 versus WT, the GO bubble (GO: 0009507, chloroplast) and (GO: 0009535, chloroplast thylakoid membrane) were in accordance with Fig 5a with the green part of GO results: there were 1749 genes in GO:0009507 and 236 genes in GO:0009535. While in Fig 5d for N3 versus WT, the GO part: red bubble (GO: 0005739, mitochondrion) and green bubble (GO:0005618, cell wall), which matched the results in Fig 5b with the GO there were 1171 genes in GO:0005739 and 218 genes in GO: 0005618 (Table S4). Fig 5e represents the top 20 GO pathways of the N2 *mut* versus WT seed transcriptome. As confirmed in the GO enrichments, N2 *mut* displayed a chalky phenotype, mainly due to significant enrichment of the related expressed genes in the chloroplasts, chloroplast thylakoid and mitochondrion, which control energy metabolism (Fig 5c and e). 

Energy metabolism is the conversion from ATP to ADP, Pi. Extracellular ATP (eATP) is involved in the (GO:0009535) pathway. In previous study, the nanomolar levels of eATP were accumulated by wounding in the plant, which triggered the ROS wave during the wounding process [30]. The cell walls of the N3 *mut* seeds are remarkably fragile, thus resulting in a very high rice broken rate, which is mainly related to the cell wall pathway (GO: 0005618). Meanwhile, the genes related to starch synthesis were down-regulated in N2 *mut*, which changed the synthesis pathway and function (Fig 7b). Moreover, these significantly enriched genes in GO are related to cell wall synthesis such as *OsCIN*, which plays a role in inducing defense in rice through sugar homeostasis regulation [31, 32], and this is similar to the results of heatmap in N3 *mut*. There were no qualitative changes in the storage of the endosperm, which implied that N3 *mut* may have a recovered mut-loci for the qualitative character but N2 *mut* does not. Meanwhile, the formation of cell walls underwent changed astonishingly, resulting in the rupture of individual cell walls under slight external mechanical pressure in N3 *mut*, which may have changed the structure of the endosperm and led to fragile phenotypes (Fig 3 and Fig S6b). 

### Venn diagram in N2/N3 mut and the volcano plots of DEGs in N2 mut

The Venn diagram shows that the DEGs were in upregulated and downregulated groups, which were based on the top 99 DEGs in the mutant seeds (3 DAP). As shown in Fig 4a of the Venn diagram, 90% of upregulated genes in N2-1 versus N3-1 *mut* groups were co-expressed genes, except for the *CSLA9* (LOC_Os06g42020). This means that all the upregulated DEGs of the mutants have been enriched in one metabolism pathway that was most active during early endosperm development. 

Comparing all the down-regulated DEGs in the overlaps of Venn diagram in mutants (Fig 4b), we found that 50% of the genes in N2-2 mutants could co-express with other mutants, while other mutant DEGs were clustered individually. There are two genes co-expressed in the overlap of N2-1 versus N2-2 mutants, *BBTI7* (LOC_Os01g033900) and *glutelin* (LOC_Os08g03410) were involved in the quality formation processes such as the regulation of energy storage and the accumulation of endogenous seed proteinases as well as thiamine. At the same time, both genes are involved in defense systems that mainly respond to biotic stresses, such as pathogens, insect bites, and mechanical damage, and they are highly expressed in flowering and early endosperm formation [33]. Through the downregulated DEGs, *aspartokinase* (LOC_Os03g63330) was co-expressed in both the N3-1 and N3-3 mutants, which was involved in the amino acid synthesis in response to drought stress [34]. The protein partner *HSP20* (LOC_Os04g36750) co-expressed in N2-1 and N3-3 mutants, was mainly involved in the activation process of the stress-induced abiotic stress response [35]. 

All of the above genes mentioned are co-expressed in the N2/N3 mutants. Overall, there were fewer co-expressed genes in the downregulated DEGs. The co-expressed genes in all of the mutants were all related to biological stress response (Table S4). One conserved protein (LOC_ Os10g11889) is a functional gene related to plant biotic stress response, and it is co-expressed in three N2 mutants (Fig 4b). Although this protein domain is highly conserved, a series of these genes, which functions under rice blast and pest stress conditions have not been reported yet [36]. *AMBP1* (LOC_Os11g37270) was co-expressed in N2-1, N2-2 and N3-1 mutants, and it could act on invasive disease by synthesis of chemicals that have a broad-spectrum and the high-efficiency resistance. However, *AMBP1* remains an unknown gene for us as its range of functions has not been reported yet. In the rice population inferred to study abiotic stress resistance, it was only found at the candidate region for salt and alkali resistance QTLs [37].*OsTPP3* (LOC_ Os07g43160) was co-expressed in N2-2, N2-z and N3-3 *mut*, which has three conserved domains to form catalytic sites for this enzyme. According to tissue-specific expression analysis, its expression level is significantly higher in vegetative flowers and seeds under the drought and salt stress treatment, and its expression level was upregulated significantly during growth and development period induce the increase of Tre6P [38].

The motif elements are involved in ABA response, which are in its promoter region include ABRE (4×) and Motif IIb (1×) elements, and the HSE (1×) and G-box (7×) motifs are involved in the heat stress and light responsiveness. In conclusion, most genes co-expressed in the Venn of mutants are involved in stimulus processes for resistance to harsh environmental stress. The trade-off balance in the energy cycles obviously affected the synthesis of the storage components of the vegetable endosperms, such as starch granules, proteinase, globulin and prolamin, which led to white-core or white-belly in the grain filling process. 

Almost all of DEGs in the volcano plot of the N2, N3 vs WT were upregulated, and the analysis results are most valuable in all RNA-seq. The top 20 significantly expressed genes were labeled (Fig 4c), 16 genes of them were related to starch synthesis, the functions of the top 5 genes significantly up-regulated in mutants are mainly concentrated on the photosynthesis, nucleus replication and energy production and transportation (Tables S7 and S8). At the same time, the top three downregulated genes are *OsNAC020* (LOC_Os01g01470), *glutelin* (LOC_Os02g15070) and *hsp 20* (LOC_Os11g13980), the downregulated genes are relative to starch metabolism, decreasing protein storage and enhancing the iron bioactivity through the membrane channels. As a result, the grain chalkiness affects quality through the presence of opaque spots in the endosperm, which was induced by affecting the starch metabolism. 

The DEGs upregulated in the Sucrose and Starch metabolic pathways in seeds (3 DAP) are related to the synthesis of soluble micro-molecular. *OsBAM2* (LOC_Os10g32810), *OsSSIII b* (LOC_Os04g53310) are the essential enzymes in the primary steps of starch degradation and synthesis, while *LTPL18-protease* (LOC_Os01g12020) was involved in lipid synthesis and pathogen invasion resistance [39]. Surprisingly, *GBSSII* (LOC_Os07g22930) is involved in the temporary starch granules transport to sink organ and *OsGLN2* (LOC_Os01g71670) involved in the 1,3-β-D-glycosidic linkages hydrophobic action of disaccharides in the cell wall, but both genes are not expressed specifically in the endosperm, but rather in the lemmas during flowering or in the germinating seeds [40]. This means that all of the genes significantly upregulated are related to energy transportation, not storage for abiotic resistance and the pathogen infection resistance (Table S9). As previous reports, the GBSSI and GBSSII were downregulated for the promotion of starch accumulation in the leaves of seedlings under the salt stress [41]. GBSSII functions as a mediating binding protein during starch granule development, which expressed specially in leaves for the transient starch in starch metabolism [42]. The top five genes significantly downregulated were *α-amylase* (LOC_Os06g49970), *GH1* (*Os3BGlu6*, LOC_Os03g11420), *GH5* (glycosyl hydrolase family 5, LOC_Os04g40510), *β-amylase* (LOC_Os03g22790), and *CPuORF23*(LOC_Os10g40550) in the DEGs of the starch synthesis pathway. *GH1*(LOC_Os03g11420) and *GH5* (LOC_Os04g40510) belong to glycosyl hydrolase, which is involved in the chemical defense, alkaloid metabolism, hydrolysis of cell wall-derived oligosaccharides, phytohormone regulation, and lignification.*CPuORF23*(LOC_Os10g40550) is involved in Trehalose synthesis which is related to drought resistance, *α-amylase* (LOC_Os06g49970) and *β-amylase* (LOC_Os03g22790) are involved in the diurnal rhythm of altering the primary carbon flux into soluble sugar for the abiotic resistance in leaves (Table S9). 

### Metabolic analysis of the Sucrose and Starch pathway in the mutant seeds (3DAP) by Mapman framework construction

**Fig. 6:**
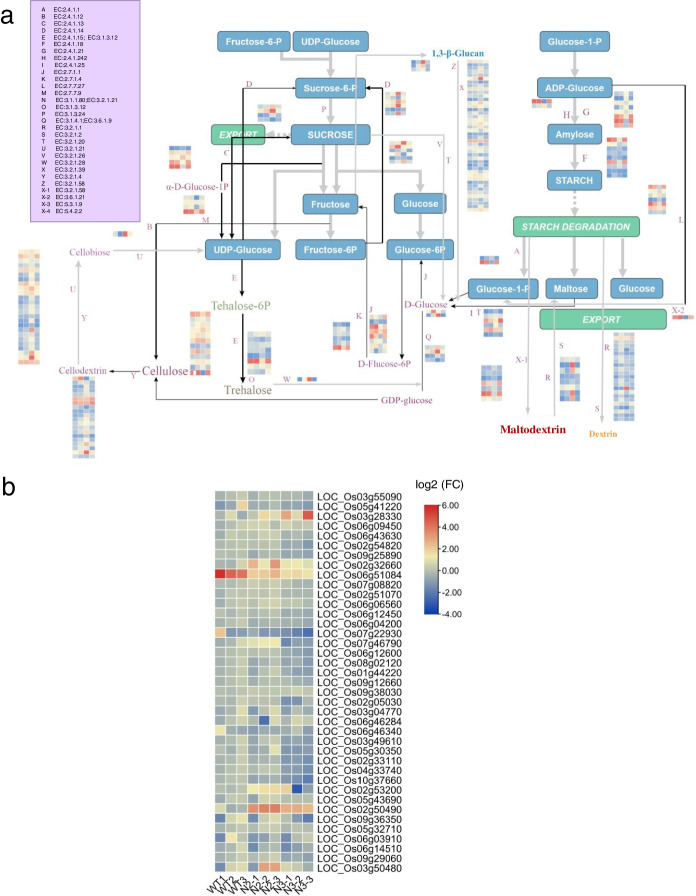
The Mapman analysis of Sucrose and Starch metabolism pathway in seeds (3DAP) of N2 vs WT and qRT-PCR verification of the DEGs in RNA-Seq KEGG. **a** The Mapman analyses are totally based on the Sucrose and Starch metabolism. The DEGs are screened from the RNA-Seq of N2 vs WT seeds (3 DAPs). There are 29 ECs in this KEGG pathway, and the heatmap diagram in each EC contain four samples: WT, n2-1, n2-2, n3-1 (Fig. S8). This Mapman constructure shows a dynamic process related to source-sink balance during grain filling procession. **b** The qRT-PCR verifications of 39 genes significantly expressed in ECs of this MapMan, which is based on the RNA-Seq KEGG in N2 vs WT seeds (3DAPs). These 39 genes expressions in seeds (3DAP) are detected repeatedly by qRT-PCR in both N2 and N3 *mut* vs WT. The almost all DEGs in mutants are down-regulated while the *RSUS1* (LOC_Os03g28330), *OsBE2b* (LOC_Os02g32660), endoglucanase 7 (LOC_Os02g50490), *OsDPE2* (LOC_Os07g46790), phosphoglucomutase 2 (LOC_Os03g50480) are up-regulated in N2 *mut* significantly (Table S11)

In Fig 6, the 29 ECs were labeled with alphabetic numbers in Starch and Sucrose metabolic pathway (ko00500). A total of 39 genes with 29 ECs were selected for the Mapman analysis of seeds in mutants versus WT. The upregulated ECs are mostly related to energy storage such as the “H and G” with the amylose synthesis, “F” with monosaccharides conversion for starch granule formation, while the ECs of starch and protein storage disintegration were downregulated, such as the “R and S” with macromolecular starch converted into disaccharides and the “F” with maltodextrin converted into maltose. However, the monosaccharides conversation between different formations was upregulated, such as “C, M, J and E” with phosphoric- or UDP- modification. Most of the DEGs in the starch and sucrose pathway were downregulated (Fig 6a). The heatmap diagram showed the qRT-PCR of 39 DEGs in Mapman KEGG in N2 mutant versus WT (Fig 6b), The different chalky phenotypes in the N2 /N3 mutant were determined by the DEGs in the starch anabolism and metabolism pathways. *RSUS1* (LOC_Os03g28330), *OsBE2b* (LOC_Os02g32660), *4-α-DPE2* (LOC_Os07g46790), *1,3-glucosidase 12*(LOC_Os02g53200), *endoglucosidase 7*(LOC_Os02g50490), and *OspPGM* (phosphoglucomutase 2, LOC_Os03g50480) were significantly upregulated in the N2/N3 mutant. Contrary, the *β-SnRK1* (LOC_Os05g41220), *SBEI* (LOC_Os06g51084) and *GBSSII* (*GBSS1b,* LOC_Os07g22930) were significantly downregulated in the mutants compared with the WT (Fig S10). Particularly in the N2 seeds (3 DAP), the AGPase large subunit 2/AGPase small subunit 1, *BAM2* and *ISA2* as the key enzymes in starch anabolism were expressed at low levels which may be the main reason for white-core formation and small round starch granules in endosperm of N2 *mut*.

Gene expression was significantly distinct in N2 versus WT: The expressions of *OsBE2b* (LOC_Os02g32660), 1,3-glucosidase 12 (LOC_Os02g53200), *endoglucosidase 7* (LOC_Os02g50490) and *OspPGM* (LOC_Os03g50480) were higher in N2 mutant (Fig 6b). Most interestingly, the expression of *RSUS1* (LOC_Os03g28330) in N3 mutant was positively higher than that in N2 mutant. 

### PPI within STRING screened co-network proteins in seeds (3 DAP) of Osnac02 mut versus WT

**Fig. 7:**
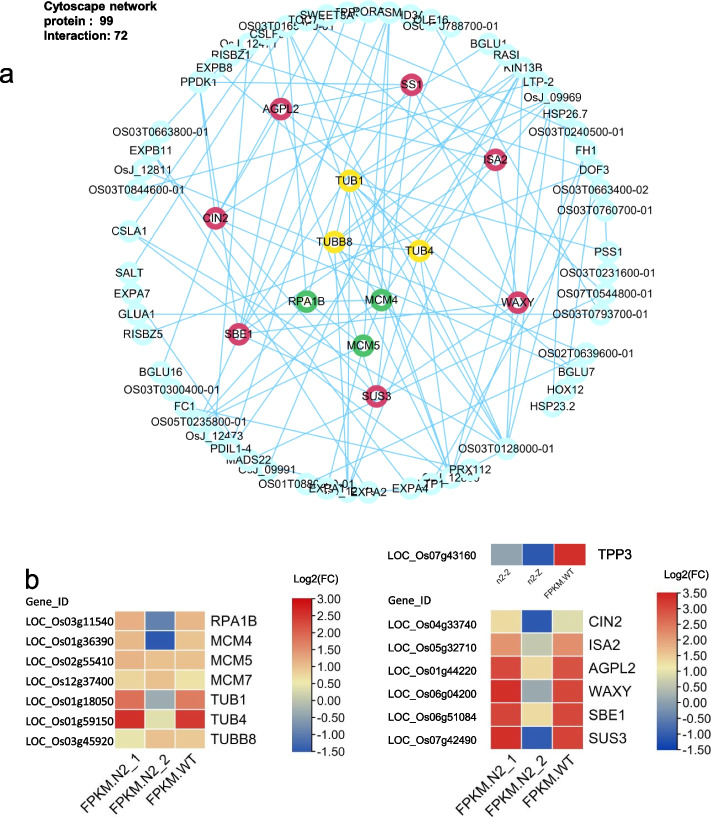
The proteins co-expressed analysis for DEGs co-network in WT vs N2 seeds (3DAPs). **a** In the RNA-seq of N2 vs WT seeds, all the pro-pro interactions representing the DEGs expressed in three co-networks. There are 99 proteins of DEGs involved in the co-network construction and only 72 proteins of them involved in the interaction, and this figure was constructed cytoscape 3.9.1. There are three groups of proteins labeled into red, green and yellow respectively with Score_threshold > 900, which are significantly co-expressed. **b** The heatmap (lg FPKM) are in accordance with DEGs expression in 3 groups of proteins which significantly co-expressed in seeds N2 vs WT, but SSI (LOC_Os06g06560) is not listed in the DEGs of the RNA-seq KEGG (Table S11)

Based on the DEGs in the RNA-seq of N2 versus WT, there were 99 proteins of DEGs involved in the co-network construction, and only 72 proteins could interact with other proteins in forming a co-expression network (Fig 7a). Of these 72 proteins, the most effective co-expressed proteins in the network were selected by the threshold (score ≥ 900), and three groups of significantly co-expressed proteins in the co-network were labeled by the red, green, yellow significantly by software (Fig 7a), as follows: GIF1 (CIN2)-GIF2 (AGPL2)-Waxy-SBEI-ISA2-SSI, Tub1-Tub4-Tub8 and MCM4-MCM5-RPA1B are significantly co-expressed as three groups (Fig 7a).What we found specially, the MCM4 and MCM5 co-network proteins were significantly enriched in both N2 and N3 mutants.

The Cytoscape prediction of protein interaction (Fig 7a) are as follows: MCM4 interacted with KIN13B which control three Tubulins directly; Meanwhile, the TPP3 interacted with SBE1 and both of them are downregulated in the Sucrose-Tre6P pathway. All of these proteins could be found in the DEGs of KEGG pathway in the RNA-seq results (Table S10). These genes composed the regulated network of starch metabolism pathway in the Ko-*Osnac02* mut. In this study, all of the seeds and materials were developed in the field of 2021 without treatment. 

Eating and cooking qualities (ECQs) are determined by various reasons and this complex characteristic was controlled by a great number of genes in the co-network [43]. And nearly all the genes in the heatmap are in a lower expression compared with WT (Fig 7b). 

In the RNA-seq of the N2 mutant seeds, the effective and marvelous DEGs and proteins in the co-network were confirmed in the early endosperm development (Table S10). In the total, there are seven genes relative to Sucrose and Starch metabolism in the co-expressed group I (Score_threshold ≥ 900), and these genes could be arranged in a classic model of Starch Synthesis (Fig S11). *AGPL 2* (LOC_Os01g44220) is a key enzyme in the primary step of the starch synthesis, and it controls the ADPG synthesis directly. This gene plays a crucial role in the regulation of starch synthesis and grain filling. *Waxy* (LOC_Os06g04200) is the key gene for Amylose synthesis, and heatmap based on the RNA-Seq KEGG of the DEGs (Fig 7b) shows that the *Waxy* expression in N2 *mut* was lower compared to WT, whereas the heatmap based on the qRT-PCR verification of Mapman KEGG shows the expression of *Waxy* (LOC_Os06g04200) and *GBSSII* (*GBSSI b*, LOC_Os07g22930) in N2 mutant was similar with that in WT (Fig S10). 

Meanwhile, in the amylopectin synthesis pathway including: *SSI* (LOC_Os06g06560), *SBE I* (LOC_Os06g51084), and *ISA2* (LOC_Os05g32710), *OsTPP3* (LOC_Os07g43160) were all downregulated (Fig 7b). SSI was not significantly expressed in the KEGG pathway, but it was expressed stably in the main step of amylopectin synthesis. *SBE I* and *ISA2* were downregulated in the DEGs of RNA-Seq KEGG, which means the amylopectin content decreasing with decreasing *SBE I* and *ISA2* expression, and then the balance between amylose and amylopectin is broken in the step of the SBE I transformation cycle (molecular amylopectin branch). As the negative feedback from the various substrates remains in the amylopectin synthesis pathway, *ADPG* will consequently flow to the amylose synthesis pathway. *OsTPP3* as the key enzyme involved in converting from Tre6P to trehalose, which responds to abiotic stress by catalyzing with Tre6P as substrates. It expressed in the vegetative seeds specially, overexpression of *OsTPP3* can enhance the drought tolerance in rice. The low expression of *OsTPP3* will result in the low level of trehalose and high level of Tre6P, which will promote the growth activity of seedling [44]. 

Integrated all the results above with that in Fig 4d, as *OsBAM2* (LOC_Os10g32810) was significantly upregulated in the amylose degradation, more amylose will be degraded as the disaccharides for developing requirement (Fig 4d). This could also be verified in the iodine-stained semi-thin sections of the opaque endosperm (5DAP) in N2 and N3 *mut* (Fig S7f and S7l)*,* and the higher amylose content in vegetative endosperm of N3 *mut* than that in N2 *mut,* this result could be verified by the darker blue shown in N3 endosperm (5DAP). *OsCIN2* (LOC_Os04g33740) is specifically expressed in immature seeds, and *gif1 (OsCIN2)* was chalky with a round smaller starch granule in the white grain [31]. In addition, *OsSUS3* (LOC_Os07g42490) corresponded to the high-temperature sensitive period during rice ripening, when its expression-level increases under the high-temperature condition. A previous study indicated that high-expression of *OsSUS3* (LOC_Os07g42490) enhances high-temperature tolerance in plants [45]. 

As the co-network proteins shown in Fig 7b, the *MCM5* (LOC_Os02g55410), *MCM4* (LOC_Os01g36390), and *RPA1B* (LOC_Os03g11540) were expressed at high levels in proliferating tissues of rice. The three genes relative to controlling the cell division in the co-network group II, affect grain shape and quality in mutants during endosperm development. 

In this study, the *MCM4*, *MCM5* and *MCM7* proteins in Prediction of Pro co-networks were significantly expressed in both N2 and N3 *mut*, which implies that these three genes may be regulated by the OsNAC06 directly. *OsNAC06* (*OsSOG1*) is the transcription factor that is homologous to *AtSOG1*(At1g26390) and regulates the cell cycle in the upstream [42]. It was found that genes regulate cell cycle including: *CYCB2.1*, *CYClazm*, *E2F2*, *MAPK*, *MCM5* (LOC_Os02g55410), *MCM4*(LOC_Os01g36390), *MCM7* (LOC_Os12g37400), *CAK1*, *CAK1A*, *CDKA1*, *CDKA2*, *CDKB*, *CYCA2.1*, *CYCA 2.3*, *CycD3*, *CDKD4*, *CDKT1*, *H1*, and *MAD2*. 

As the stranded DNA binding complex subunit 1, *RPA1B* (LOC_Os03g11540) is a one replication protein, positively expressed in proliferating tissues such as the root tips and young leaves, which contain root apical meristem and marginal meristem respectively [43]. In eukaryotes, the MCM family of proteins plays an important role in chromosome replication process. The normal DNA replication in every cell cycle is accurately controlled by binding the origin recognition complex that recruits MCM proteins including MCM2 - MCM7, which are highly conserved in organisms [46, 47]. The MCM complex consists of six different subunits (MCM2 - MCM7), and it interacts to form a ring-shaped hetero-hexamer with a central channel large enough to encircle the DNA. Once the MCM complex has been loaded, origins are licensed to replicate and any site containing the MCM complex has the potential to form an active DNA replication fork [46]. In human beings, *MCM4* expression decrease about 5% will cause the DNA damage response [48]. In Arabidopsis, the subunits of the MCM2- MCM7 complex were coordinately expressed during the development stage, which is abundant in proliferating and endocycling tissues. This displays the indicative role of DNA replication in plants, the *MCM5* and *MCM7* subunits remain in the nucleus during the S and G2 phases of the cell cycle, whereas *MCM4*, *MCM6*, and *MCM7* form a “core” complexes, which are more tightly associated with chromatin than the remaining subunits [47]. 

The three Tubulins such as *OsTUB1* (LOC_Os01g18050), *OsTUB4* (LOC_Os01g59150) and *OsTUBB8* (LOC_Os03g45920) were interactive in the co-network group III. There are α-tubulins and β-tubulins in the microtubule family, which are strictly conserved in protein coding regions of almost all eukaryotes. There are eight TUB isotypes with 27 predicted amino acid sequences of *TUBs* in rice [49]. 

Tubulin protein is reported to be expressed in the dividing tissues [50], which may play roles in most of the cell division processes and cell elongation [51]. However, in a previous study, the tubulins (TUBB) are verified to be expressed tissue-preferentially, and they are positively expressed in dividing tissues, including root tips and tender leaves. However, the reduced expression of *AtTUA6* (α-tubulin) in Arabidopsis has been shown to affect root growth and morphogenesis severely [52]. *OsTUB4* (LOC_Os01g59150), which is expressed stably in all tissues, is used as the internal control for qRT-PCR in almost all plants [53], while the *OsTUB1*(LOC_Os01g18050) function has yet to be elucidated. Meanwhile, as an anther-specific β-tubulin, *OsTUBB8* (LOC_Os03g45920) is also expressed at high levels in vegetative growth, such as anthers and seed set in rice [51]. Because these Tubulins are highly conserved in DNA sequences and play an essential role in cellular processes, their low-level expression may also affect the most active division process - the grain filling. 

## Discussion

There are two phases in a plant growing period: the vegetative phase with germination and plant growth, the reproductive phase with flowing and spikelet fertility. During the process of plant growth, sugar signals play an important role through the Sucrose and Starch metabolic pathway. The glycolysis dependent on SnRK1/TOR signal transduction pathways is a crucial glucose signal transduction pathway in plants [54]. 

For SnRK1/TOR signaling transduction pathway indirectly responds to glucose signaling by sensing energy consumption and environmental stress in plant, which control the plant growth and development [55]. The Tre6P-Sucrose transportation between source organ such as leaf and sink organ such as seed, root. *OsNAC02* as TF which involved in the DNA replication and cell cycle control, and the variation of its expression could result in the SSC fluctuation. Changes in these processes can induce a shift in the energy metabolism system, leading to the formation of floury endosperm at early development of seeds with 50% white-core, and the Ko-mutants are all premature as a result. 

### Ko-mutants prematurity relative to chalky phenotype and quality change in the vegetative endosperm development

The seeds of all mutants mature about 15 days earlier than those of WT, and display the yellowish glumes in mutant and green caryopses in WT (95 DAG). Meanwhile, there is a great qualitative variation in the endosperm of mature and dry seeds in mutants versus WT. As the SSC is up-regulated significantly in the mutant seed (125 DAG) (Fig S7), the reason of premature may be that the sugar is a key bio-signal for triggering the vegetative development, and a higher SSC in seed will enhance this signal including the sucrose, maltose, glucose, which regulated these processes on mRNA-level and post-mRNA-level by miR156 [56]. Integration of volcano Plots and Mapman results with Prediction of protein interaction, the relative genes in the Starch and Sucrose metabolism pathway (Figs 4d and 6b), such as *OsBAM2* (LOC_Os10g32810) and *OsSSIIIb* (LOC_Os04g53310) in the volcano-DEGs (Fig 4d), the *RSUS1* (LOC_Os03g28330), *OsBE2b* (LOC_Os02g32660), *OsDPE2* (LOC_Os07g46790) in the Mapman KEGG, *GBSSII* (LOC_Os07g22930), *OsGLN2* (LOC_Os01g71670) in Venn analysis, which results primary changes in the quality of mutant seeds, the energy transformation from storage amyloplast to the chloroplast (Fig S11). SSC was upregulated and the DP ≤ 13 of amylopectin were obviously increased in the N2 mut, the chalkiness with white-core in mutant seeds induced through the amylopectin syntheses increasing, all of these phenotypes above have been reported previously [57, 58]. These upregulated genes can induce chalky in endosperm and variations in physicochemical properties. 

The *OsBE2b* (LOC_Os02g32660) overexpressed in N2 mutant which may have a slight increase in short amylopectin chains (DP=10-12), and this result is similar to that of an accumulation of excessive branched, water-soluble polysaccharides in OE-*BEIIb* mutant [59].This Physiochemical properties just like the RS, which is a small fraction of starch resistant to hydrolysis by exhaustive α-amylase and PUL *in vitro*.*RSUS1* is presented in the aleurone of the developing seed and plays a role in sugar transport into endosperm cells [60, 61], but in the Ko-*Osnac02* mutant where the *RSUS3* was down-regulated in endosperm, which could increase the sucrose in seed. *OsBAM2* (LOC_Os10g32810) located in the chloroplast was up-regulated in N2 mutant, and it could increase the maltose content and inducing excessive amylopectin decreased into a balance [62]. Meanwhile, *OsDPE2* (LOC_Os07g46790) responds to the maltose increasing stress and transport the maltose to other glycogen, in order to decrease the maltose ratio in seeds [63]. As the soluble sugar in endosperm increased, the juvenile to adult phase is shortened, and the resistance to abiotic stress is increased in mutant as well. *OsGLN2* (LOC_Os01g71670) encode an enzyme endo-1,3-b-glucanase (1,3-b-GLU), which is expressed in the germinating seed [64]. It belonged to a huge family named glycosyl hydrolases family17 in plants, involving in the transportation of sugar onto a lipophilic acceptor which changed its chemical properties, and alters the bioactivity, enabling access to membrane transporter systems [65, 66]. The glycosylation of *OsGLN* is important for improving the resistance of abiotic stress such as salt stress in rice [67]. 

In the early stage of endosperm development, the amylose content in seeds (5 DAP) of N2 mutant was significantly lower compared to N3 mutant (Fig S7f), which is contrary to the result that the amylose content in N2 mutant seeds (125 DAG) significantly decreased to a lower level at mature stage (Fig 3b). This result may be induced by the increased expression of *OsSSIIIb* (LOC_Os04g53310) in the endosperm and *GBSSII* (LOC_Os07g22930) in the chloroplast, which means more energy was stored in leaf instead of being transported to the sucrose-sink in endosperm, and there were more amylopectin and less amylose in N2 seeds compared to WT and N3. 

Combining the DEGs in Prediction of Pro interaction and Mapman KEGG based on seeds transcriptome (Fig 6b), all the key genes down-regulated are including *β-SnRK1* (LOC_Os05g41220), *SBEI* (LOC_Os06g51084), *OsSUS3*(LOC_Os07g42490) and *OsTPP3* (LOC_ Os07g43160). While *OsCIN1*(LOC_Os02g33110), *OsCIN2* (LOC_Os04g33740), *AGPL2* (LOC_Os01g44220), *ISA2* (LOC_Os05g32710) are expressed in no difference between N2/N3 and WT. 

The *SBE I* (LOC_Os06g51084) are specially expressed in the filling grains, and mainly for synthesis of the medium and long amylopectin chains. The decreased expression of SBE I in Ko-*Osnac02* mutant may show a slight decrease in long amylopectin chains (DP > 37) and intermediate chains (DP =12-21), while a slight increase in short amylopectin chains (DP < 12) [68], but no significant change in the appearance and weight of the caryopsis are found as the results in Fig 3d [69, 70]. Both the *SBE I* and *OsTPP3* was down regulated in the Sucrose and Starch pathway as well as the *β-*SnRK1, and the two genes have a interaction in the Prediction of Pro network (Fig S11). This may be the reason for high Tre6P production [55], which will induce the premature and white-core in endosperm. 

SnRK1 complex composed of α1, α2, β, γ subunits is the central metabolic regulator in the sucrose-dependent precession, and it is specific in the plant which is regulated by variations of sucrose content. *β-*SnRK1 (LOC_Os05g41220) together with γ-SnRK1 are two regulatory subunits of SnRK1 complex [71], this gene down-regulated in N2 *mut* can inhibit the sucrose transferring to ethanol and glycerol in cytoplast [72]. Meanwhile, the high content of sucrose stimulates Tre6P accumulation will inhibit the SnRK1 expression in the Suc-Tre6P nexus model [54, 73], this means that *SnRK1* down-regulated could promote the resources being used for reactivating growth and reproduction in order to balance high content of Tre6P (Fig 6b). *OsSUS3* (LOC_Os07g42490) is named Rice Sucrose Synthase 3 for its expression in rice only, and predominantly in the seed endosperm where it played an important role in starch filling processes during the milky stage of rice seed ripening [74]. It has been selected as a key enzyme in the sucrose-to-starch conversion of rice spikelet during the grain filling under abiotic stress, and its overexpression enhances the thermo-tolerance in rice [45, 75]. As the first key enzyme in sucrose synthesis, the down-regulated expression of *OsSUS3* might feedback to the high sucrose content. Overall, all of these gene expressions in the starch synthesis in seeds (3 DAP) were downregulated during early endosperm development. This caused the smaller starch granules in the central of the endosperm, and the amylose and amylopectin contents of the mature seeds (125 DAG) were lower compared with WT. 

### DEGs in Ko-mutants are relative to abiotic resistance in the vegetative endosperm

Integration of the volcano plot and Venn analysis in Fig 4 and the mapman KEGG in Fig 6b, and most of these genes are relative to enhancing abiotic stress resistance. Such as endoglucanase 7 (LOC_Os02g50490), *OspPGM* (LOC_Os03g50480), *OsVTC2* (LOC_Os12g08810), *OsBAM2*(LOC_Os10g32810), *OsSSIIIb* (LOC_Os04g53310), *GBSSII* (LOC_Os07g22930), *LTPL18* (LOC_Os01g12020), *OsGLN2* (LOC_Os01g71670), *glutelin* (LOC_Os02g15070), *ycf68* domain (LOC_Os04g16722) are up-regulated in the DEGs. And most of these genes were relative to abiotic stress in N2 mutant. 

Endoglucanase 7 (LOC_Os02g50490) was highly expressed in N2/N3 *mut*, and involved in cell wall synthesis and cell wall organization process under abiotic stress in roots [76, 77]. As these genes are highly up-regulated in N2 *mut*, which mainly promote the starch degradation and the soluble sucrose production, as well as enhance the cell resistance to abiotic stress. The *OspPGM* (LOC_Os03g50480) which significantly up-regulated in the N2 mainly related to the sterility of male pollination, and expressed highly at the starch synthesis stage [78]. This gene could respond to abiotic stress by sucrose-signal as well (Fig S9b). *OsGLN2* which is primary expressed in the endosperms of germinating seeds where its expression is induced by GA and suppressed by ABA [64]. *OsVTC2* (LOC_Os12g08810) encodes the rate-limiting enzyme in the ascorbate synthesis pathway, and the plant with higher ascorbate content enhances the salt stress tolerance and Fe bioavailability [79]. These genes above are highly upregulated in N2 *mut*, and they mainly promote starch degradation and the soluble sucrose production, as well as enhance cell resistance to abiotic stress. 

According to the down-regulated DEGs in the volcano (Fig 4c), the most prominent genes are as follows: the *ONAC020* (LOC_Os01g01470), *GH1* (Os3BGlu6, LOC_Os03g11420), *GH5*(LOC_Os04g40510), *OsCPuORF23* (LOC_Os10g40550), *β-amylase* (LOC_Os03g22790), *OsTPP3* (LOC_Os07g43160). In this study, *GH1* (LOC_Os03g11420) and *GH5* (LOC_Os04g40510) as the *β-glucosidases* are expressed in the germinating seed or the leaves as cellulose tissues involving in carbohydrate metabolism in the vegetative development, which inhibit the seed filling rate. Their expression could be induced by the transient starch in KEGG pathways, and also be activated in the transient starch transportation [9, 80]. *CPuORF23* (LOC_Os10g40550) involved in trehalose synthesis, which is relative to drought resistance [34]. All these genes deletion can reduce the cell walls resistance to abiotic and biotic stress and delay the production of disaccharide as a substate transfer to cell walls synthesis. 

In conclusion, premature development of rice grains can be triggered by multiple factors. Dehydration induced by heat stress and moderate drought stress play significant roles, along with ER stress. Additional under the drought stress for 15 days, several OsBAM(β-amylase) will up-regulated in leaves, which suggesting that starch was mainly metabolized by degradation rather than synthesis during the drought stress [6]. In this study, the *OsHSFA2s* are obviously overexpressed in leaves (Fig 1d), this means the ER-stress was up-regulated for abiotic resistance [81, 82].

### Formation of chalky seeds by incompletely filling in the vegetative endosperm

According to the Prediction of Pro co-network in seed (3DAP), three groups of co-networks genes were selected on the threshold (score ≥ 900), and seven genes of group I control the chalkiness formation in the early endosperm development, including: *Waxy* (LOC_Os06g04200), *OsAGPL2* (LOC_Os01g44220), *SSI* (LOC_Os06g06560), *SBE I* (LOC_Os06g51084), *ISA 2* (LOC_Os05g32710) (Fig S9b), besides with *ONAC020* (LOC_Os01g01470) (Fig. 4c).

In the whole starch metabolism pathway, *AGPL2* (LOC_Os01g44220), *SSI* (LOC_Os06g06560), *SBEI* (LOC_Os06g51084), *ISA2* (LOC_Os05g32710) are active in amylopectin synthesis, which are down-regulated in N2 seed (3DAP) mutant (Fig 6c). Meanwhile, *Waxy* (LOC_Os06g04200) is active in amylose synthesis with a stable expression at normal-levels. In the previous study, knock-out or down-regulated these genes can induce chalkiness in rice endosperm. 

As ADP-glucose pyrophosphorylase (AGP) large subunit that plays a crucial role in the regulation of starch biosynthesis and grain filling during rice seed development, the deletion of *OsAGPL2* and *OsAGPS2b* causes a shrunken endosperm due to a significant reduction in starch synthesis [83–86]. Through the yeast-two-hybrid assay, *OsAGPL2* interact with *OsAGPS1*, *OsAGPS2a* and *OsAGPS2b,* the expressions of granule-bound starch synthase, starch synthase, starch branching enzyme and starch debranching enzyme are dominantly decreased in *gif2* (OsAGPL2) mutant [87]. 

Waxy and dull rice grains have an opaque endosperm due to the pores between and within the starch granules [88]. *Waxy-I* bears a loss-of-function mutation that results in glutinous rice varieties with extremely low AC, *Waxy-II* shows a leaky phenotype with a moderate level of AC, and *Waxy III* functions as WT allele with high AC [89]. 

As homologous genes *ISA1* and *ISA2* have similar functions in the amylopectin precursor formation process in rice endosperm. FLO6 (CBM48) could interact with *ISA1* directly to participate in the relative starch protein folding process, and plays the same role as *SSG4* in controlling the size of starch granules, then the floury endosperm can be formed by controlling the size of the compound granules [4, 90]. 

In previous study of chalky rice, *OsNAC20* or *OsNAC26* caused starch and storage protein synthesis decreasing in their mutants. When the mutation of *OsNAC20* or *OsNAC26* occurs alone with on change in grains, but the *OsNAC20/26* double mutant could significantly decrease the starch and storage protein content. Because they could bind to the promoters of *AGPS2b*, *AGPL2*, and *SBEI*, and the expression of these three genes was severely decreased in the *OsNAC20/26* mutant [91]. As the *OsNAC020* (LOC_Os01g01470) is down-regulated in the early filling stage (Fig 4c), then the storage of starch and protein decrease and the expression of *AGPL2*, *SBEI*, *Waxy* was consequently downregulated in the starch pathway (Fig 6c). 

Overview all the genes above in the starch synthesis in seeds (3DAP), the most gene expressions were down-regulated during the early development. This causes the starch granules smaller in the central of endosperm, and the content of amylose and amylopectin in mature seeds (125 DAG) was lower than that of WT. According to the Prediction of Pro STRING data of interactive DEGs in RNA-seq based on the N2 *mut* seeds (3DAP) versus WT, the *OsSUS3* expression is down-regulated in the endosperm, however, the *RSUS1* in the elongated cell such as the aleurone is up-regulated. This may be the reason why both white-core and white-bell are formed in N2 seed, and this expression model may be an urgency strategy used for harsh environment resistance. Because the endosperm development was omitted while the aleurone, caryopses development of seeds in a normal rate, then the floury endosperm formed as a result, and this may be why the SSC content in N2 *mut* are much higher in mature seeds (125 DAG) compared with WT. 

In conclusion, according to the qRT-PCR verification of Mapman KEGG in starch synthesis, 90% of genes in the ECs were down-regulated (Fig S9b). The Starch is the main component of the rice embryo, which occupied 76.7 % - 78.4 % of fresh seed and 95 % weight in dry seed, while the storage proteins occupy only 8 % of the dried rice embryo. The different ratios of amylose and amylopectin combinations contribute the different quantities of rice embryos. In the early endosperm development, due to insufficient starch synthesis, the soluble substrates content such as amylose and soluble sugar was higher for the necessary energy up-conversion, followed by a chalky phenotype with a white-core and white-belly in the endosperm. 

### Premature caryopses in N2 mut with cell cycle shorting in dividing cells

Cell cycle controlling genes such as the MCM family, Tublin proteins, NAC family are mainly expressed in dividing tissues, and their subcellular locations are mostly in the nucleus. Their expressions change with the DNA replication, and they are conserved in the domains of the protein structures in different plants. 

The MCM family of proteins play an important role in the chromosome replication process in eukaryotes [46], and MCM2-MCM7 are highly conserved in organisms. In the process of chromosome replication, the MCM DNA protein plays a role in stabilizing chromosomes during the cell cycle G0-G1, which was responsible for the precise replication of the chromosomal DNA. At the same time, the MCM is involved in the formation and stabilization of centrioles during mitosis. It was demonstrated that when the MCM expression decreased in the G0-G1 phase, it would lead to DNA damage and chromosomal abnormalities, activate the expression of ATR/ATM gene and induce expression of CHK1/CHK2 [48]. 

In animals, any reduction in the expression of MCM4 also lead to DNA abnormalities. In Fig 6, as the PPI shown, KIN13B which is the key enzyme in binding ATP was regulated by the MCM4, and the KIN13B directly regulated TUB1, TUB4, TUBB8 simultaneously. Meanwhile, we found that *MCM4* (LOC_Os01g36390) showed a low-expression in N2 *mut*, and the TUB1, TUB4 are down-regulated in N2 mutant (Fig 6b). MCM4 as the core subunit of the MCM2- MCM7 complex in animals, and the deletion of MCM4 may form a special minichromosome-loss phenotype which can induced apoptosis and death with homozygosity by homosomal abnormalities [92].This result may be the reason for the shortage of grain filling period: First, the MCM4 expression was indirectly down regulated with *OsNAC02* low expression in the Ko-*Osnac02* mutant. Second, the KIN13B regulated genes *TUB1*, *TUB4* with a lower expression in N2 *mutant*, which may indirectly control by MCM4. 

## Conclusion

In the *OsNAC02* co-network based on DEGs of N2 versus WT (Fig S11), the effective genes regulated by OsNAC02 are mainly TFs, and they respond to both heat stress and other abiotic stress for the multiple functional motifs in their promoter regions. OsNAC06 is mainly involved in regulating cell cycle changes and speeding endosperm filling rates under the environmental stress; all of these processes are controlled by a series of genes expressed in the nucleus, which determine chromosome replication. The down-regulated expression of these genes, such as MCMs and TUBBs, accelerates the replication cycle and rate, which also affects energy storage and consuming rate. 

From the DEGs in the volcano plot (Fig 4d), the Mapman overview on Sucrose and Starch Metabolic pathway (Fig 6), the Prediction of Protein co-network (Fig 7), 50% of the crucial genes in ECs are collected by statistical analysis (log2 FC, *p* < 0.05). The important enzymes in the pathway are including: BAM2, SSIIIb, GBSSII, RSUS1, pPGM were upregulated, as well as CIN2(GIF1), AGPL2(GIF2), WAXY, ISA2 are expressed with no significance, and SBEI was downregulated in N2 mut. The regulation of these genes promoted micromolecular production such as sucrose, whereas the macromolecular storage of substrates decreased during the early seed-filling stage. All of these results above could cause floury endosperm in mutants. It is confirmed that the decreased AC in the N2 mut endosperm, as well as the SSC increasing significantly in N2 *mut* seeds (125 DAG) are relative to the upregulated expression of *RSUS1*(LOC_Os03g28330), *OspPGM* (LOC_Os03g50480) (Fig 6b) and *OsBAM2* (LOC_Os10g32810) in the DEGs of N2/N3 mutant versus WT (Fig 4c). In the Ko-*Osnac02 mut*, a decreased energy storage induced the soluble sucrose content increasing, which promotes faster development and enhances the resistance to the biotic and abiotic stresses in the whole plant. In full-filled grains, *OsNAC02* deletion will affect the morphological characters including a decrease in the quality of endosperm and an acceleration in seed development period obviously. Because OsNAC02 could interact with more than five TF proteins simultaneously, and its mRNA is unique in the upstream suggests that these genes may all regulate cellular processes through a specific mechanism. Therefore, any effective change in the expression of these TFs can lead to energy imbalance and activation of the phytohormone pathways. From these results, we find that even a small change in *OsNAC02* expression may indirectly affect the important pathways associated with cell division. 

## Materials and methods

### The construction of Ko-Osnac02 and Ko-Osnac06 mutants

Both N2 and N3 *mut* are multiple mutants and be constructed using the Crispr-Cas9-Vector. Homozygote transplants were obtained from T1 and T2 generations in 2020 and 2021 separately. The mutants were constructed by the Ku-Cas9-pl-Ko-vector (Kan^+^), with tags designed on the CDS of gene sequences with NGG/NAG as PAM-seq (Fig. 1a and b). The tags in the Crispr-Cas system are designed by software online (http://www.biogle.cn/index/excrispr) or (http://crispr.dbcls.jp/). In order to detect the function of OsNAC02, the Ko-mutant transplants (T0) were obtained and the expression of relative genes are detected by qRT-PCR (Fig. 2a and d).

### The yield characters detection and growth conditions

The Nipponbare (*Oryza sativa*, ssp. *japonica*) was used for Knockout mutant construction including the N2 *mut* (*Osnac02*), N3*mut* (*Osnac02/Osnac06*) by Crispr-Cas9*.* All the materials were harvested as T2 at the summer of 2021. The shape phenotypes such as length, width and thickness are detected on the seeds at 95 DAG and T1 generation at 125 DAG in the winter of 2020, respectively. 

First, the materials are grown in the fields without any treatment, and every mutant are planted in a plot with 5 x 3 lines arrangement. The transplants of T0 generation were planted in Lingshui, Hainan Province (18.5°110’E) at the winter in 2020, where we got T1 generation of mutant. Then transplants of T1 generation are planted in Fuyang, Hangzhou, Zhejiang Province (30°120’E) during the summer of 2021, and the T2 generation was obtained with the yield characters of mutants collection at the autumn in 2021. After drying in the for 60 days, the yield characters are detected including number of seeds per panicle, yield per plant, the weight of 1000-grains per plant. In addition, five randomly selected panicles per plant are used for yield characters detection, such as panicle length, numbers of panicles per plant and number of grains per panicle (Fig S1). All of the important botanic and agronomic characters of the T2 mutants (95 DAG) are detected as follows: the plant height, tiller number, the primary branches and the available panicles number per plants (Fu Yang, Hang Zhou, 2021). 

The caryopses were separated from grains using a small milled machine, and brown rice was selected from a random group of five panicles per plant for measurement of yield characteristics, The results were obtained using an automatic seed testing analyzer with 1000-grain weight meter (Model Type: Wanshen SC-G seed testing instrument). The experimental materials are including: N2 (*Osnac02-1*) and N3 (*Osnac02/Osnac06-1*), the WT (Nipponbare) was used as the control. 

### The homologous analysis and the locations of motif in promoter of TFs regulated by OsNAC02

Homologs of *OsNAC02* and *OsNAC06* were identified through a BLASTP search settings in the GenBank. These sequences were extracted from databases of NCBI and Uniprot. The similarity of NAC protein sequences homology was analyzed using the DNAMAN 7.0 (Fig S4a). The phylogenetic tree of NAC family in rice with the Neighbor-Joining cluster was constructed using the MEGA 7.0 (Fig S3). 

In co-network of *OsNAC02*, motifs of TFs with abiotic resistance containing ABRE/G-box we screened in the promoters of OsMYB4, OsHSFA2c and OsHSFA2e on affyPLM method, which was used for quality control and producing the genes expression matrices. 

 (https://www.bioconductor.org/packages/release/bioc/html/affyPLM.html). The results are listed in Fig S5a. All the motif locations on the promoters of TFs are detected by the PlantCARE online (http://bioinformatics.psb.ugent.be/webtools/plantcare), and the mimic graph of model for the motifs locations were displayed using the software IBS 1.0 (Fig S5b). 

### The relative expression in T1 seedlings by qRT-PCR

In 2021, the total RNA was isolated from the specific tissues of T1 generation (95 DAG), such as root, stem, sheath, flag leaf and seeds (3 DAP) were directly collected from the field. The total RNA of these tissues was extracted from these tissues using TRIzol (Invitrogen, CA, USA) for reverse transcription to generate the plant total cDNA. Relative quantification variation between replicates was measured by (△-△CT) method. For qRT-PCR, the house-keeping gene ubiquitin (LOC_Os03g13170, mRNA accession number AK100267) was used as an internal control. Three biological replicates were performed, and the primers of qRT-PCR are listed in Table S12. 

### The yeast-one-hybrid assay

The interactions of OsNAC02 regulated genes in the PPI was verified by the yeast-one-hybrid (*Saccharomyces cerevisiae*, Y-1-H) assay which was belong to the Wan academicians’ laboratory. This system consists of *pB42AD*-vector as the active part and *pLacZi*-vector as the effective part. The CDS of *OsNAC02* was cloned and ligased with the *pB42AD* vector (EcoR I), and the promoter sequences (-2000bp -‘ATG’) of the relative genes are linked with the *pLacZi* vector (Xho I), including *pOsMYB04*, *pOsHSFA2E* and *pOsHSFA2C*. The positive control was constructed by the reference information relative to the key genes *OsNAC02*(LOC_Os04g38720) in ABA pathway [24]. The vector transformation and screening procedures were followed the protocols established in ‘WanJianmin lab’ (accession number AK100267), which was used as an internal control [3]. The primers for all these vectors’ constructions are designed by PrimerPremier 6.0 (Table S1). 

### A series detection of the quality characters and phenotypes of brown rice in N2/N3 mutant

The detected phenotypes include the shape of brown rice such as grain length, grain width, grain thickness, and the chalky detection of milled rice per plant includes chalky ratio, chalky area, the rice broken ratio. All the characters are evaluated according to the standards of key lab in CNRRI. For the phenotype detection, three samples were randomly selected from 100g seeds. The chalky characters during endosperm development of the Ko-mutant were also detected through the semi-thin sections observations and hand-cut sections in floury endosperms (3 DAP and 5 DAP).

### Physicochemical properties detection in milled rice

The different mutant grains were dehydration at room temperature (28°C-30°C) in our lab for 60 days and milled grains are used for the detection of the percentage of chalky grains (PCG), percentage of chalky area (PCA) and percentage of endosperm chalkiness (PEC). We also detected the physicochemical properties, such as protein content, the ratios of total starch, amylase (AS) /amylopectin (ISA), the starch chains length (DP), and SSC including sucrose, glucose, fructose. the student *t-*test (*p* < 0.05) was used to calculate the physicochemical properties of milled rice between the mutants and wild type.

### Cellular observation

The observation of starch granules in the central part of transverse section was conducted using scanning electron microscopy (SEM). To observe the starch granules in mature endosperm, the cross-sections of mature caryopses were sputter-coated with gold and the semi-thin sections of the opaque endosperm were observed under the scanning electron microscope (SEM). The grain samples were prepared by direct dehydration to a critical point with liquid CO2, mounted on SEM stubs and then coated with the gold palladium. The mounted specimens were observed and photographed under the SEM. 

The samples of endosperm development observation were fixed with a formaldehyde-acetic acid-ethanol fixative solution and stored at 4°C, then dehydrated in a gradient ethanol series and infiltrated with xylene for embedding in resin for the preparation of histological analysis. The hand-cut sections of filling endosperm of 3 DAP, 5 DAP, 8 DAP, 10 DAP were sliced in 2.0 um-5.0 um-thick sections. The cellular observation of endosperm development stained with Lugol’s iodine: 0.02% iodine (I_2_) and 0.2% potassium iodide (I_2_-KI), and then the sections were observed and photographed under a bright-field microscope (Olympus). The pre-treatment of all the samples was conducted as follows: the filling samples at different stage were sectioned transversally into semi-thin-slides, immersed in staining liquid for 30 seconds, and then dried these slides on the 37°C. 

In summary, the cell walls and starch granules of endosperm observation are using transmission electron microscopy (TEM), allowing for the observation of cell volume, shape and numbers in the same scope area. Meanwhile, the endosperm structure was observed using semi-thin sections with scanning electron microscopy (SEM). Physicochemical changes in the central part of T2 endosperm were detected by electron microscopy (TEM) and iodide staining (I_2_-KI) observations in 2022. 

### The total RNA extraction and library construction for RNA-seq

The total RNAs of panicles (3DAP) were extracted using the total RNA Mini-prep kit (Axygen, http://www.axygen.com) and the PrimeScript Reverse Transcriptase kit (TaKaRa, http://www.takara-bio.com). The quality of total RNA was detected by NanoDrop ND-1000 (NanoDrop, Wilmington, DE, USA). The isolation of total RNAs was using the DNA kit. According to the manufactures’ instructions including two rounds of amplification using RNA extraction kit named Message Amplification II R (Thermo fisher, https://www.thermofisher.cn/).

RNA-Seq libraries were pooled and sequenced on the platform of lllumina Novaseq TM 6000 (illumine, http://www.illumina.com/). All the sequencing database were performed as raw data according to manufacturer’s instructions using the Illumina Novaseq TM 6000 control. 

### Muti-genomic analysis for co-network construction

The RNA-Seq analysis was based on seeds (3 DAP) of T2 mutants planted under no treatment in 2021, and biological replicates for N2/N3 mutant were used for the data verification by PCA analysis (Fig S8). The raw data was generated from the illumine-seq machine and then aligned to the reference rice genome MSU.7.0 (http://rice.plantbiology.msu.edu.). The raw data was filtered based on the designated criteria to the Rice Genome Annotation Project, which clustering samples that have significant *P*-values, so as to make sure the normalized data qualified. 

After deleting redundant sequences from raw data, DEGs were used for constructing heatmaps and Venn diagrams, especially the genes relative to control grain quality are selected. We performed the actor screening significant DEGs and identified by qRT-PCR. The heatmap diagrams need the log_2_FC to be more than 0.5, this could be verified by the quantitative PCR analysis and another format of DEGs expression. 

The PCA, volcano plots and Venn diagrams are based on classification of gene expressions, and the GO enrichment and KEGG pathway analysis are based on the expression pattens of DEGs between mutants and WT. All these visualized analysis of RNA-seq are completed on the online platform of LC-BIO company (http://www.lc-bio.com). Additionally, all the diagrams related to the basic analysis of RNA-seq are constructed by the software TBtools 11.02 [93]. 

In this study, the Mapman framework of Starch and Sucrose metabolic pathway was constructed, however, the results display in a new way. As all the DEGs are based on the KEGG pathways, the ECs in pathway are used for the hierarchal allocated DEGs in functional categories of Mapman. The expression of DEGs was performed through Heatmap and qRT-PCR for each ECs in the Mapman KEGG pathway (Fig S9b) [94]. The primers of DEGs are designed by PrimerPremier 6.0 for qRT-PCR verification (Table S12). 

The PPI analysis are performed by “annotate your proteome to STRING” section on the internet, which can be used for the transcription factors such as OsNAC02, and its regulated co-network proteins as well. The Cytoscape 3.9.1 software was used to shown 99 expressed co-network proteins [95]. The proteins in Prediction of Pro subgroup with DEGs in mutants versus WT (Threshold_score > 900) are selected for STRING construction (STRING, https://cn.string-db.org/), and all the networks created by STRING can be transported to Cytoscape online. Submit the proteome of DEGs, STRING will output the interaction network and predict the protein functions (including GO terms, KEGG pathways). 

### Accession numbers

Gene Bank: *OsNAC02*, LOC4329852, OSNPB_020594800, LOC_Os02g38130; 

*OsNAC06*, LOC4340715, OSNPB_060267500. 

RAP_Locus : *OsNAC02*(Os02g0594800), *OsNAC06*(Os06g0267500) ; 

Sequence data from this article can be found in the GenBank/EMBL, Processed RNA-seq

data can be accessed and visualized at NCBI, and the platform website: http://www.lc-bio.com/

The gene ID in RNA-seq is accordance with MSU_loc **(**Rice Genome Annotation Project (uga.edu)**)**

*OsNAC02*(LOC_Os02g38130), *OsMYB04*(LOC_Os04g43680), *OsHSFA2E* (LOC_Os03g58160), 

*OsHSFA2C*(LOC_Os10g28340), *OsHSFB2c*(LOC_Os09g35790); 

*Glutelin* (LOC_Os02g15070), *α-amylase* (LOC_Os06g49970), *OsBAM2*(LOC_Os10g32810); 

*SSI*(LOC_Os06g06560), *OsVTC2* (LOC_Os12g08810), *endoglucanase7* (LOC_Os02g50490), 

*RSUS1* (LOC_Os03g28330), *OspPGM* (LOC_Os03g50480), *OsBE2b* (LOC_Os02g32660), 

*OsDPE2* (LOC_Os07g46790), *OsGLN2* (LOC_Os01g71670), *glutelin* (LOC_Os02g15070), 

*ycf68* domain (LOC_Os04g16722), *OsSSIIIb* (LOC_Os04g53310), *GBSSII* (LOC_Os07g22930), 

*LTPL18* (LOC_Os01g12020); 

*MCM4* (LOC_Os01g36390), *MCM5*(LOC_Os02g55410), *MCM7*(LOC_Os12g37400), 

*OsTUB1* (LOC_Os01g18050), *OsTUB4* (LOC_Os01g59150), *OsTUBB8*(LOC_Os03g45920), 

*RPA1B*(LOC_Os03g11540); 

*β-SnRK1* (LOC_Os05g41220), *SBE1* (LOC_Os06g51084), *CIN2*(LOC_Os04g33740), *AGPL2*

 (LOC_Os01g44220), *ISA2*(LOC_Os05g32710), *OsSUS3* (LOC_Os07g42490); 

*OsNAC20* (LOC_Os01g01470), *OsPUP2* (LOC_Os09g29210), *OsTPP3* (LOC_Os07g43160), 

*BBTI7* ( LOC_Os01g03390), Aspartokinase (LOC_Os03g63330), *Hsp20* (LOC_Os04g36750), 

*CPuORF23* (LOC_Os10g40550); 

*Os3BGlu6* (LOC_Os03g11420), *GH5* (LOC_Os04g40510), *ZmEBE-1* (LOC_Os01g26320), 

*GDSL* like synthase (LOC_Os01g46220), *CSLA9* (LOC_Os06g42020). 

The qRT-PCR verification of 39 genes in 29 ECs in Starch and Sucrose metabolic pathways are selected from DEGs of Mapman analysis in RNA-Seq, and the Accession Numbers in details are listed in Table S12. 

### Supplementary Information

**Additional file 1:** **Figure S1.** The mutant tags of target sites and mutants detection by PCR sequencing. **Figure S2.** The yield characters of T2 N2 /N3 *mut* (95 DAG) in field (95 DAG in 2021, 125 DAG in 2020). **Figure S3.** The homologous clusters of OsNAC family genes by phylogenetic analysis. **Figure S4.** The homologous analysis of NAC in plant. The Gene ID are according to the database RAP (rapdb.dna.affrc.go.jp), and (*OsNAC02*, Os02g0594800), (*OsNAC06*, Os06g0267500) in NCBI of homologous analysis. **Figure S5.** The ABRE/ G-box motif locations on the promoter regions of OsNAC02 regulated genes. **Figure S6.** The chalky phenotype of N2/N3 *mut* seeds (T2) in 2021. **Figure S7.** The histological analysis during endosperm development. **Figure S8.** The PCA clusters based on RNA-seq analysis. **Figure S9.** The heatmap diagram of DEGs in N2/N3 versus WT. **Figure S10.** The genes of 39 ECs in Sucrose and Starch metabolic pathway in Mapman of N2 vs WT seeds (3DAP) are verified by qRT-PCR. **Figure S11.** The antagonism between energy storage and development requirement under stress in Ko-*Osnac02* mutant.

**Additional file 2:** **Table S1.** The H-1-Y vectors construction of genes in OsNAC02 co-network. **Table S2.** The data of WTVSN2_YVSN2_Z_Gene_differential_expression notation. **Table S3.** The data of WTVSN3_1VSN3_3_Gene_differential_expression notation. **Table S4.** The GO enrichment analysis of N2 vs WT and N3 vs WT respectively. **Table S5.** The genes co-expressed in the down-regulated Venn diagram (N2 vs N3 vs WT, Fig. 4b). **Table S6.** The up- and down-regulated genes notation of Venn diagram in seed (3DAP). **Table S7.** The data of the WTVSN2_XVSN3_Y DEGs in Volcano diagram. **Table S8.** The volcano diagram of genes labeled in DEGs of the seeds (N2 vs N3 vs WT, Fig. 4c). **Table S9.** The volcano plot of labeled genes in Sucrose and Starch pathway of the seeds (N2 vs N3 vs WT, Fig. 4d). **Table S10.** The DEGs in the Prediction of Protein interaction of WTVSN2_xVSN2_y_ for heatmap. **Table S11.** The qRT-PCR primers of DEGs in starch and sucrose pathway ECs for Mapman verification. **Table S12.** The qRT-PCR of genes in co-network of OsNAC02.

## Data Availability

The raw data of rice genome as the reference or control in RNA-Seq have been deposited in the Genome Sequence Archive (Genomics, Proteomics & Bioinformatics, 2021). The raw data of RNA-Seq in this manuscript have been deposited in the National Genomics Data Center (https://ngdc.cncb.ac.cn/gsa/, Nucleic Acids Res 2022), the China National Center for Bioinformation Beijing Institute of Genomics, Chinese Academy of Sciences (GSA: CRA008460), which are publicly accessible online (https://ngdc.cncb.ac.cn/gsa/browse/CRA008460). The RNA libraries were sequenced on the illumina Novaseq™ 6000 platform by LC Bio Technology CO., Ltd (Hangzhou, China) and obtained with the help from Dr. Peng. The data of physicochemical properties detections are obtained according to the procedures in our lab, and you could contact Dr.Wei (weixiangjin@caas.cn) for the details of the detection methods.
