# Supplementary material for: Chalkiness and premature controlled by energy homeostasis in OsNAC02 Ko-mutant during vegetative endosperm development
Source: BMC Plant Biol. 2024 Mar 18;24:196. doi: 10.1186/s12870-024-04845-8 (PMC10946104; doi:10.1186/s12870-024-04845-8)
Supplement: Supplementary file 2 — Additional file 2: Table S1. The H-1-Y vectors construction of genes in OsNAC02 co-network. Table S2. The data of WTVSN2_YVSN2_Z_Gene_differential_expression notation. Table S3. The data of WTVSN3_1VSN3_3_Gene_differential_expression notation. Table S4. The GO enrichment analysis of N2 vs WT and N3 vs WT respectively. Table S5. The genes co-expressed in the down-regulated Venn diagram (N2 vs N3 vs WT, Fig. 4b). Table S6. The up- and down-regulated genes notation of Venn diagram in seed (3DAP). Table S7. The data of the WTVSN2_XVSN3_Y DEGs in Volcano diagram. Table S8. The volcano diagram of genes labeled in DEGs of the seeds (N2 vs N3 vs WT, Fig. 4c). Table S9. The volcano plot of labeled genes in Sucrose and Starch pathway of the seeds (N2 vs N3 vs WT, Fig. 4d). Table S10. The DEGs in the Prediction of Protein interaction of WTVSN2_xVSN2_y_ for heatmap. Table S11. The qRT-PCR primers of DEGs in starch and sucrose pathway ECs for Mapman verification. Table S12. The qRT-PCR of genes in co-network of OsNAC02. [file 12870_2024_4845_MOESM2_ESM.pdf]

**a** Table The tags of target sites designed for *Osnac02* /*Osnac06* mutants

| tag | site | Tag sequence (PAM) |
|---|---|---|
| *Osnac06* | | |
| Tag1 | 231-253 | CACAAAGTTTGGCGGTAGTGCGG |
| Tag2 | 1256-1278 | CCTCATCGCAAGATCCGAAATGG |
| *Osnac02* | | |
| Tag1 | 399-421 | GGAAGACTGGCGGGTTAAGACAG |
| Tag2 | 1344-1366 | GGAGTAACGAGATCAGCGGGTTA |

**b-1** **Os*nac02*** **Tag** 399-421 bp (-strand)

| | | | | | | | | | | | | | | | | | |
|---|---|---|---|---|---|---|---|---|---|---|---|---|---|---|---|---|---|
| **Tag** | **G** | **G** | **A** | **G** | **T** | **A** | **A** | **C** | **G** | **A** | **G** | **A** | **T** | **C** | **A** | **G** | **C** |
| Wt | C | C | T | C | **A** | **T** | **T** | **G** | **C** | **T** | **C** | T | A | G | T | C | G |
| mut | C | C | T | C | - | - | - | - | - | - | - | T | A | G | T | C | G |

**Os*nac02*** **Tag** 1350-1360 bp

| | | | | | | | | | | | | | | | | | |
|---|---|---|---|---|---|---|---|---|---|---|---|---|---|---|---|---|---|
| **Wt** | **C** | **A** | **G** | **A** | **T** | **G** | **G** | **A** | **A** | **T** | **G** | **T** | **C** | **C** | **A** | **A** | **A** | **C** |
| mut | C | A | G | A | T | G | G | **C** | A | T | G | T | C | C | A | **T** | - | C |

**b-2** **Os*nac02*** **Tag** 399- 421 bp (-strand)

| | | | | | | | | | | | | | | | | | | | | | | |
|---|---|---|---|---|---|---|---|---|---|---|---|---|---|---|---|---|---|---|---|---|---|---|
| **Tag** | **G** | **G** | **A** | **G** | **T** | **A** | **A** | **C** | **G** | **A** | **G** | **A** | **T** | **C** | **A** | **G** | **C** | **G** | **G** | **G** | **T** | **T** | **A** |
| **Wt** | C | C | T | C | A | T | T | G | C | T | C | T | A | G | T | C | G | C | C | C | **A** | T | **T** |
| **mut** | C | C | T | C | A | T | T | G | C | T | C | T | A | G | T | C | G | C | C | C | **A** | - | **T** |

**Os*nac06*** **Tag** 1256-1278 bp (-strand)

| | | | | | | | | | | | | | | | | | | | | | | | |
|---|---|---|---|---|---|---|---|---|---|---|---|---|---|---|---|---|---|---|---|---|---|---|---|
| **tag** | **C** | **C** | **T** | **C** | **A** | **T** | **C** | **G** | **C** | **A** | **A** | **G** | **A** | **T** | **C** | **C** | **G** | - | **A** | A | A | **T** | **G** | **G** |
| **Wt** | G | G | A | G | T | A | G | C | G | T | T | C | T | A | G | G | C | - | **T** | T | T | A | **C** | **C** |
| **mut1** | G | G | A | G | T | A | G | C | G | T | T | C | T | A | G | G | C | T | **T** | T | T | A | **C** | **C** |
| **mut2** | C | C | T | C | A | T | C | G | C | A | A | G | A | T | - | - | - | - | **T** | T | T | A | **C** | **C** |

**c**

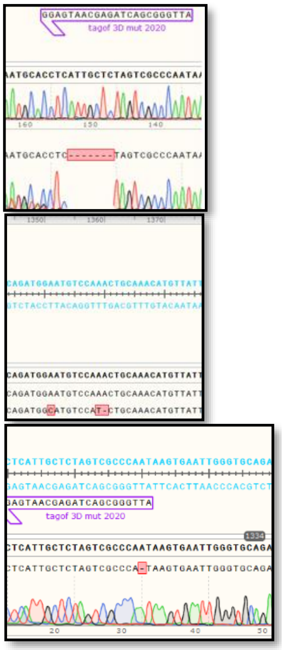


**Fig.S1** The mutant tags of target sites and mutants detection by PCR sequencing

**(a)** The tags of target sites designed for *Osnac02* /*Osnac06* mutants, including the PAM sequences and locations.
**(b-1)** Ko-*Osnac02 mut* construction with two mutant sites in *OsNAC02* CDS. The genetic type of mutants are labeled in red and the PAM "GGA" is highlighted in bold and underline. The mutant with "ATTGCTC" 7 bp-deletion in N2 *mut* located at 399-421 bp of *OsNAC02* sequence, and the mutant with "A→C" at 1350 bp, " A→T " / " A→x " transversion at 1360 bp of *OsNAC06* sequence.
**(b-2)** N3 *mut* constructed the mutant sites in *OsNAC02 / OsNAC06* CDS with PAM "GGA"/ "TGG" respectively. The Crispr-Cas9 induced "T" deletion in N2 and "T" insertion and 3 bp -"GGC" deletion in N3 at 1256-1278bp region respectively.
**(c)** As these figures shows, the mutant sites of N2/N3 *mut* in *OsNAC02* CDS detected by the alignment with DNA sequence of WT (NIP).

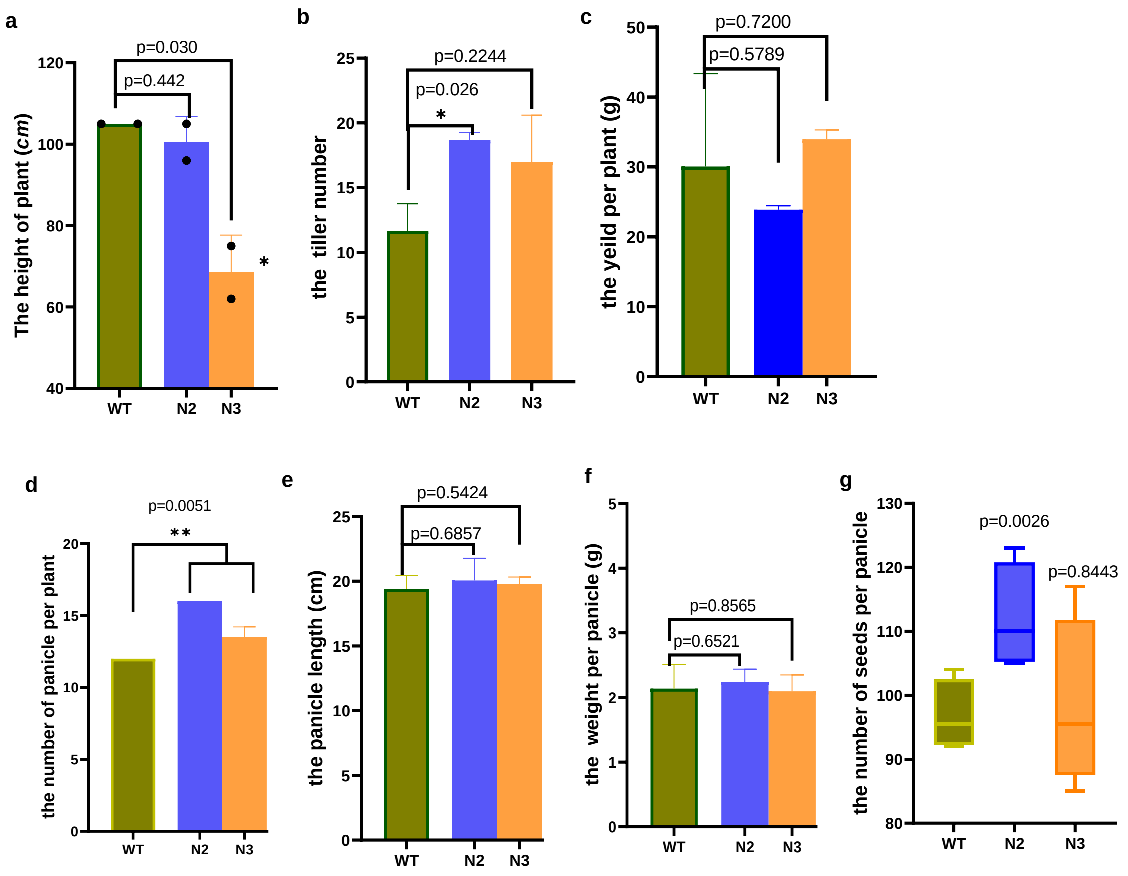


**Fig.S2** The yield characters of N2 / N3 *mut* in field (95DAG in 2021, 125DAG in 2020)

**(a)** The plant height of N2/N3 mutant (T1) shows obvious difference in 2021, and both of them were dwarf compared with the WT. The height of N3 is higher than N2 mutant.
**(b)** The number of tillers in N2 and N3 mutant are more compared with WT in 2021.
**(c)** The N2/N3 mutant (T0) harvested in Hainan are detected for the yield per plant. All the seeds of T1 generation (125 DAG) are pre-treated with drying in the green house for two months. The yield per plant in N3 *mut* was heaviest and N2 *mut* was the lightest as a result.
**(d)** The number of panicles per plant (95DAG) in N2/N3 mutant, and the plant height, the tiller number per plant are detected simultaneously in 2020.
**(e)** The panicle length per plant (95DAG) in N2/N3 mutant was detected in 2021.
**(f)** The weight per panicles (95DAG) of N2/N3 mutant are detected, and there is no significant difference between mutants and WT. Five panicles from each plant are selected randomly for detection respectively.
**(g)** The number of seeds per panicle in T1 generation (95DAGs) was detected, and N2 *mut* has most seeds per plant. This phenotype together was detected with the number of panicles together in the same materials.

All the mutants planted in field under the normal conditions (Fuyang, Hangzhou in 2021),the statistic analysis are running by the student *t-test*,*, $p < 0.05$ and **, $p < 0.01$. The T0 generation of transplants was planted in Lingshui (Hainan Province Island ,18.5°110'E) in the winter of 2020, and we obtained the T1 generation. Following the T1 generation was planted in Fuyang (Hangzhou in Zhejiang Province, 30°120'E) during the summer in 2021, and T2 generation were harvested in the autumn of 2022.

As the yield characters shown in **Fig 1**, the shape phenotype in seeds (125 DAG) of N2/N3 mutants are no difference compared with the control (NIP).

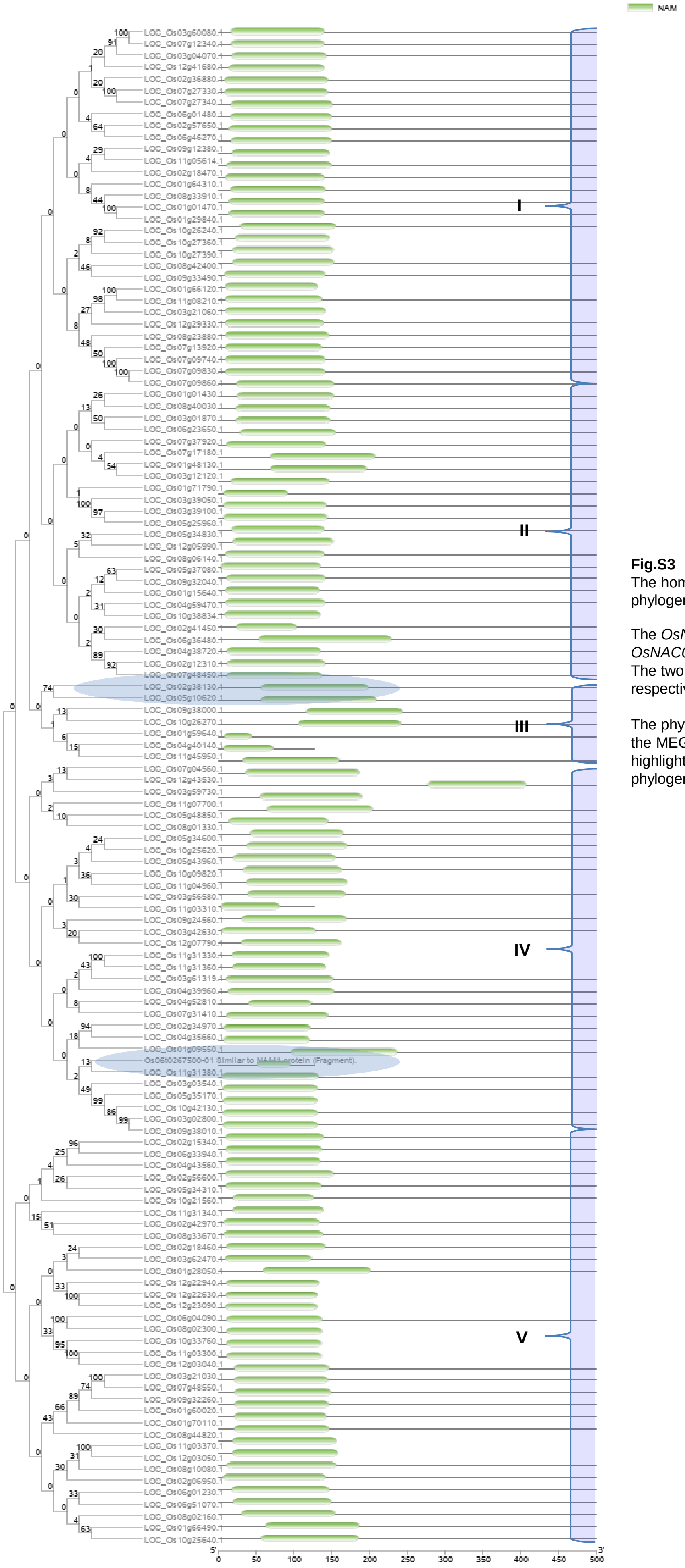


**Fig.S3**
The homologous clusters of OsNAC family genes by phylogenetic analysis

The *OsNAC02* (*Os02g0594800*, *LOC_Os02g38130*) and *OsNAC06* (*Os06g0267500*) are labeled by bule circle. The two genes are belonged to subgroup III and IV respectively.

The phylogenetic tree of OsNAC family was constructed using the MEGA 7.0. and the NAM regions in all genes are highlighted by TBtools11.2. The green boxes in the phylogenetic tree are NAC domains of genes.

**a**

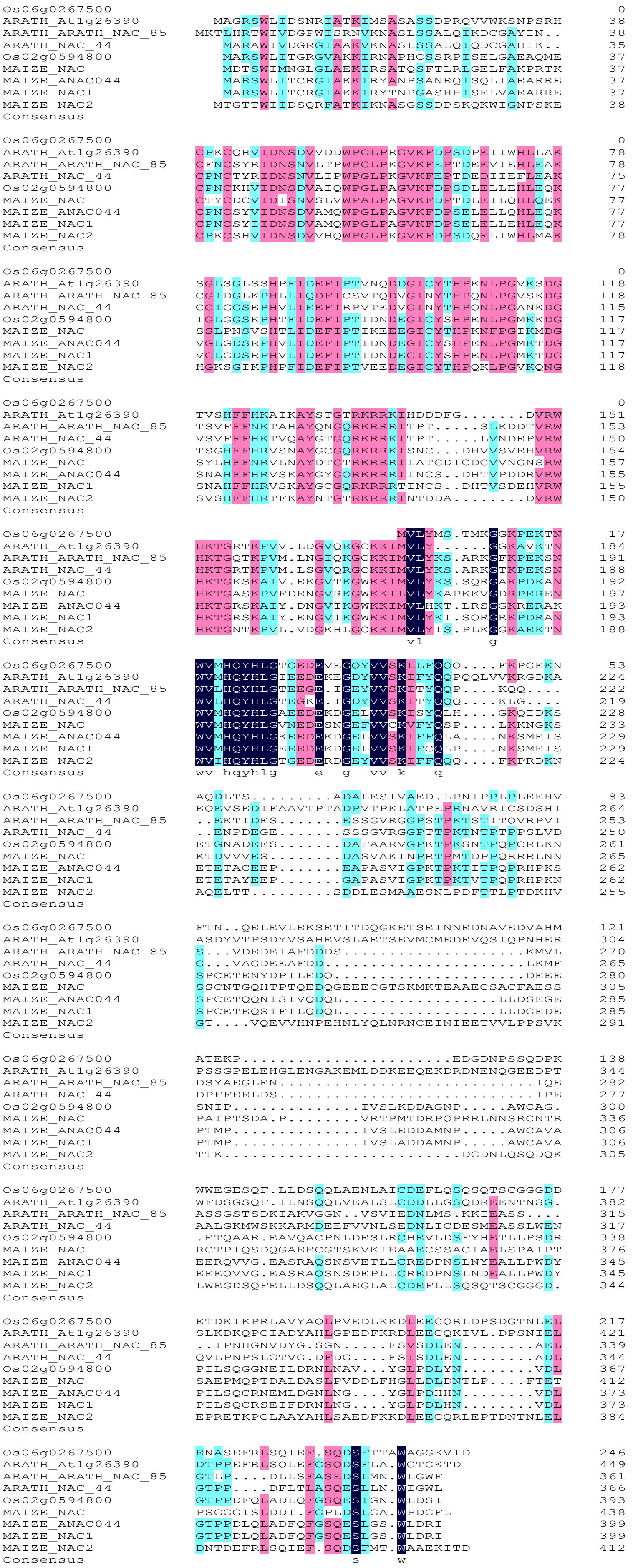

**b**

| Name | Pro-aa | Gene ID | Protein entry |
|---|---|---|---|
| **OsNAC02** | 393 | Os02g0594800 | Q6ZI65_ORYSJ |
| **Zem-NAC** | 438 | Zm00014a_036995 | A0A3L6FRI6_MAIZE |
| **ANAC044** | 399 | Zm00001e022870 | B6TBS0_MAIZE |
| **NAC1** | 399 | NAC1 | C0PGC9_MAIZE |
| **NAC2** | 412 | ZEAMMB73_Zm00001d037315 | A0A1D6LWF7 |

| Name | Pro-aa | Gene ID | Protein entry |
|---|---|---|---|
| **OsNAC06** | 246 | Os06g0267500 | Q5Z9V2_ORYSJ |
| **AtSOG1** | 499 | At1g26390 | A0A178WLI1 |
| **ARATH_NAC085** | 361 | At5g13980 | A0A178UA43 |
| **NAC_44** | 366 | At3g00730 | A0A178VDA1 |

**Fig.S4** The homologous analysis of NAC in plant

**(a)** The homologous analysis of NAC proteins with amino acid sequence alignment in the rice, maize and Arabidopsis by using DNAMAN 7.0.

**(b)** The Uniprot accession of 9 genes in homologous analysis. (https://www.uniprot.org/). *OsNAC06* (*Os06g0267500*) has simple protein constructure and contains only 246 aa, which was highly homologous to *AtSOG1*. Its N-terminal region with 0 - 45aa was a strictly conserved region of NAM domain.

**a**

**Table** the motif analysis of genes regulated by OsNAC02 in Co-network

| Gene | ID | Motif | Motif seq | start | end |
|---|---|---|---|---|---|
| OsNAC02 | LOC_Os02g38130 | —— | —— | —— | —— |
| OsNAC06 | Os06g0267500 | ABRE4 | CACGTA | 838 | 844 |
| OsMYB04 | LOC_Os04g43680 | ABRE | AACCCGG | 166 | 171 |
| | | ABRE | ACGTG | 1559 | 1552 |
| | | G-Box | CACGTT | 165 | 159 |
| OsHSFA2C | LOC_Os10g28340 | ABRE/G-box | CACGTG | 1079 | 1073 |
| OsHSFA2E | LOC_Os03g58160 | ABRE | GACACGTATG | 1947 | 1939 |
| | | ACE | GACACGTATG | 1947 | 1957 |

**b**

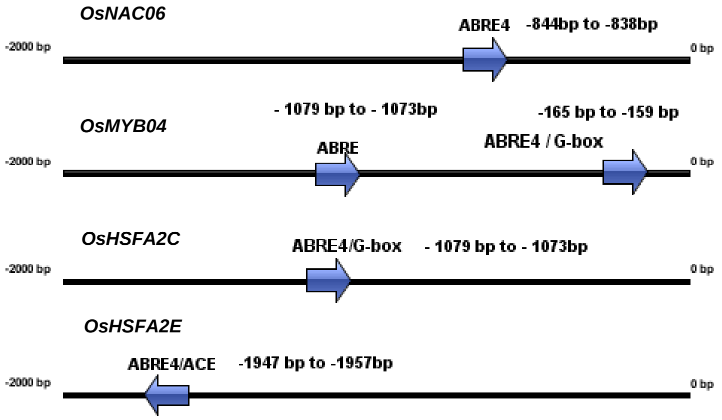


**Fig.S5** The ABRE/ G-box motif locations on the promoter regions of OsNAC02 regulated genes

**(a)** The motif analysis of genes regulated by OsNAC02 in co-network, all the location are detected by the PlantCARE. (http://bioinformatics.psb.ugent.be/webtools/plantcare).

**(b)** The model of the motif analysis in the promoter region (-2000bp-'ATG') of the OsNAC02 effective genes, as the arrow shows the direction of motifs with (+/-) on the promoter and the fig construction was completed by the software IBS1.0.

a

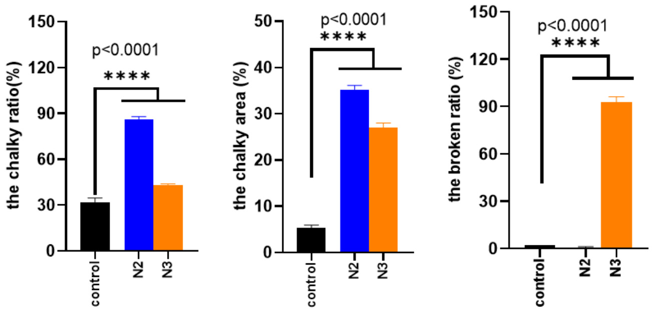


b

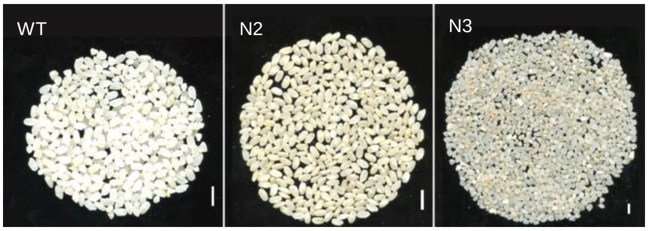


c

**Table** Chalky phenotype of mutants by the statistics analysis (%)

| Characters | WT | N2（*Osnac02*） | N3（*Osnac02 /Osnac06* ） |
|---|---|---|---|
| Chalky ratio ( Number ) | 12% | 75% | 12.6% |
| Chalky ratio (area) | 5% | 30% | 10% |
| rice broken ratio | 2% | 0% | 90% |

**Fig.S6** The chalky phenotype of N2/N3 *mut* seeds (T2) in 2021

**(a)**The chalky phenotypes of the milled rice are calculated by chalky ratio and the chalky area . The transparent are low in both mutant and WT, but the chalky ratio in mutants are obvious higher than that in WT. The rice broken ratio of N3 *mut* is highest of all. All the statistic analysis were performed by running a two-way ANOVA on the mixed model, *, $p < 0.05$, **, $p < 0.01$ and ***, $p < 0.001$.
**(b)** The sample names are WT, N2 and N3 in the figure respectively. As the chalky phenotypes of milled rice and broken ratio of N2 and N3 *mut* per plant shown in this figure, both N2 and N3 are core-white in milled rice, but only N2 has core and belly white in its floury endosperm. As these figures show the yield per plant simultaneously, only N3 has highest yield and broken ratio in the milled rice per plant. n=10 mm.
**(c)** The three phenotypes of transparent in N2 and N3 mutant by statistics analysis.

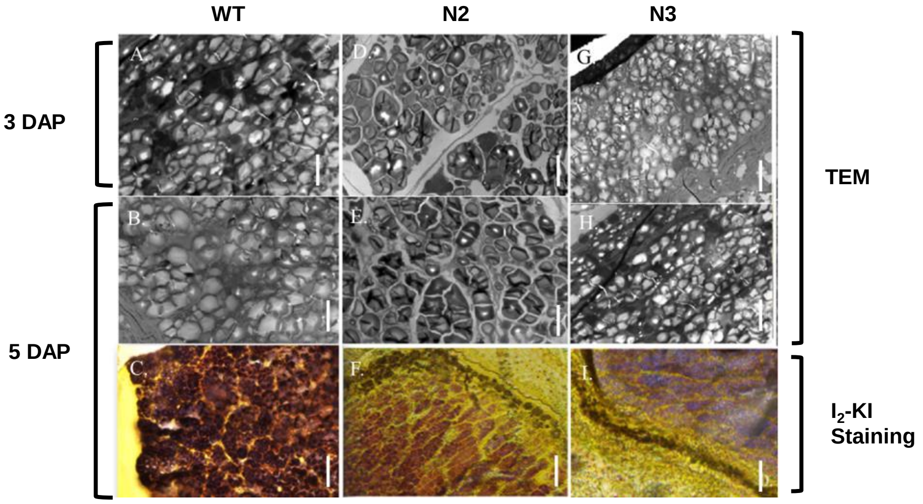


**Fig.S7** The histological analysis during endosperm development

Histological analysis of endosperm for maturation detection. As above shows the semi-thin sections of the opaque endosperm scanning in TEM and KI-$I_2$ stains. n=100 um.
The processes in endosperm development of WT/N2/N3 are shown in this figure respectively. As the **fig c**, **fig f**, **fig I** show the semi-thin sections stained of opaque endosperms at 5 DAP by the $I_2$-KI.The iodine-staining(**$I_2$-KI**) semi-sections implied the distinct starch content between N3 *mut* and N2 *mut*. For a darker blue in N3 *mut* (5 DAP) implies there are higher Amylase content in N3 *mut* (**fig I**) than that in N2 *mut* (**fig f**).
**Abrreviation:** TEM, transmission electron microscope; DAP, days after pollination.

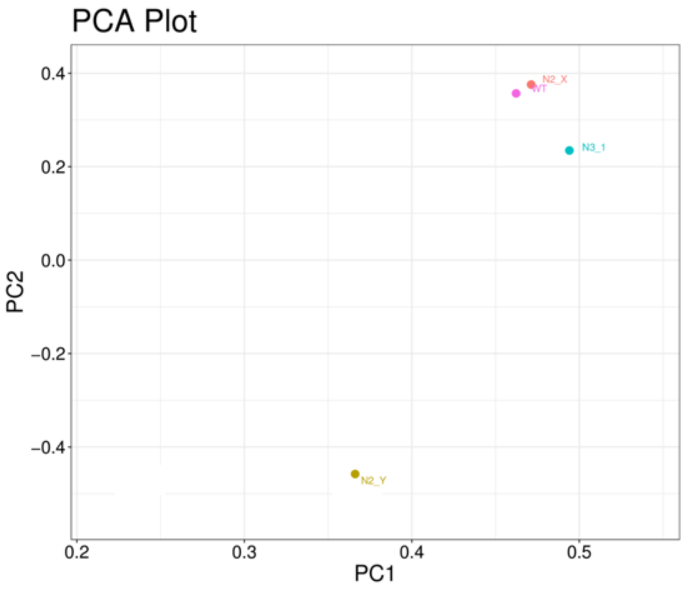


**Fig.S8** PCA clusters based on RNA-seq analysis

The trend of PCA analysis are based on the RNA-seq analysis of seed (3 DAP),and four samples are clustered into 3 PCA groups:
The red spot was PCA1 cluster with WT and N2-1 (N2-x);
The yellow spot was PCA2 cluster with N2-2 (N2-y) ,which used as duplicate sample of N2-x for the KO-*Osnac02 mut* ;
The blue spot was PCA3 cluster with N3_1 only.

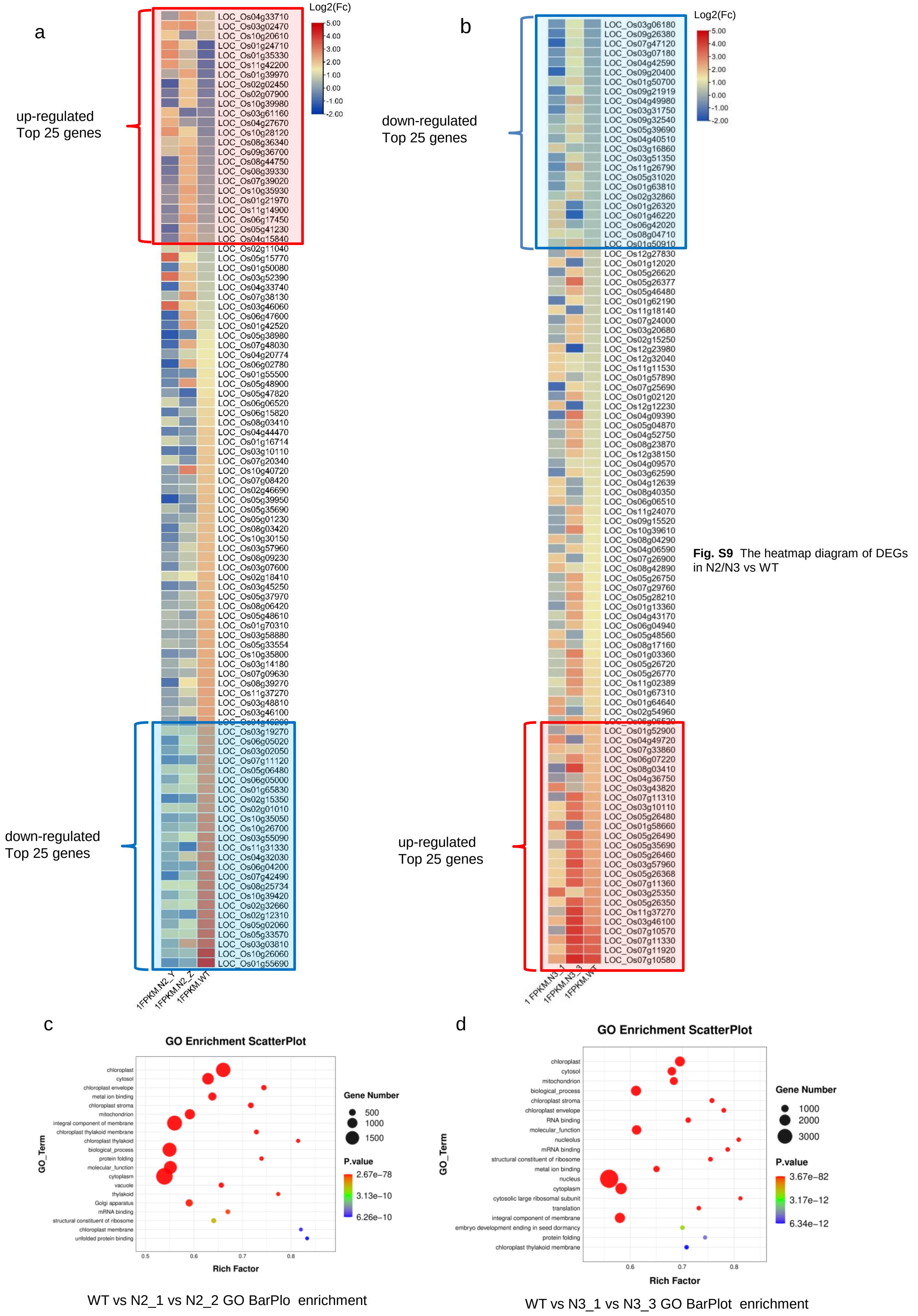


**Fig. S9** The heatmap diagram of DEGs in N2/N3 vs WT

**Fig.S9** The heatmap diagram of DEGs in N2/N3 versus WT

**(a)** The distinct expressed genes (DEGs) in the heatmap of **N2 vs WT** in an ascending ordering ;
**(b)**The distinct expressed genes (DEGs) in the heatmap of **N3 vs WT** in an ascending ordering, all the RNA-seq data were standardized by the log10 parameter, and the top 25 genes with highest expression are highlighted in the red frame ,and the top 25 genes with lowest expression are outstanding in the blue frame;
**(c)** The GO analysis of the **N2 vs WT** was according to the top 20 GO enrichment pathways of **N2-1 vs N2-2 vs WT** ;
**(d)** The GO analysis of the **N3 vs WT** was according to the top 20 GO enrichment pathways of **N3-1 vs N3-2 vs WT**

.

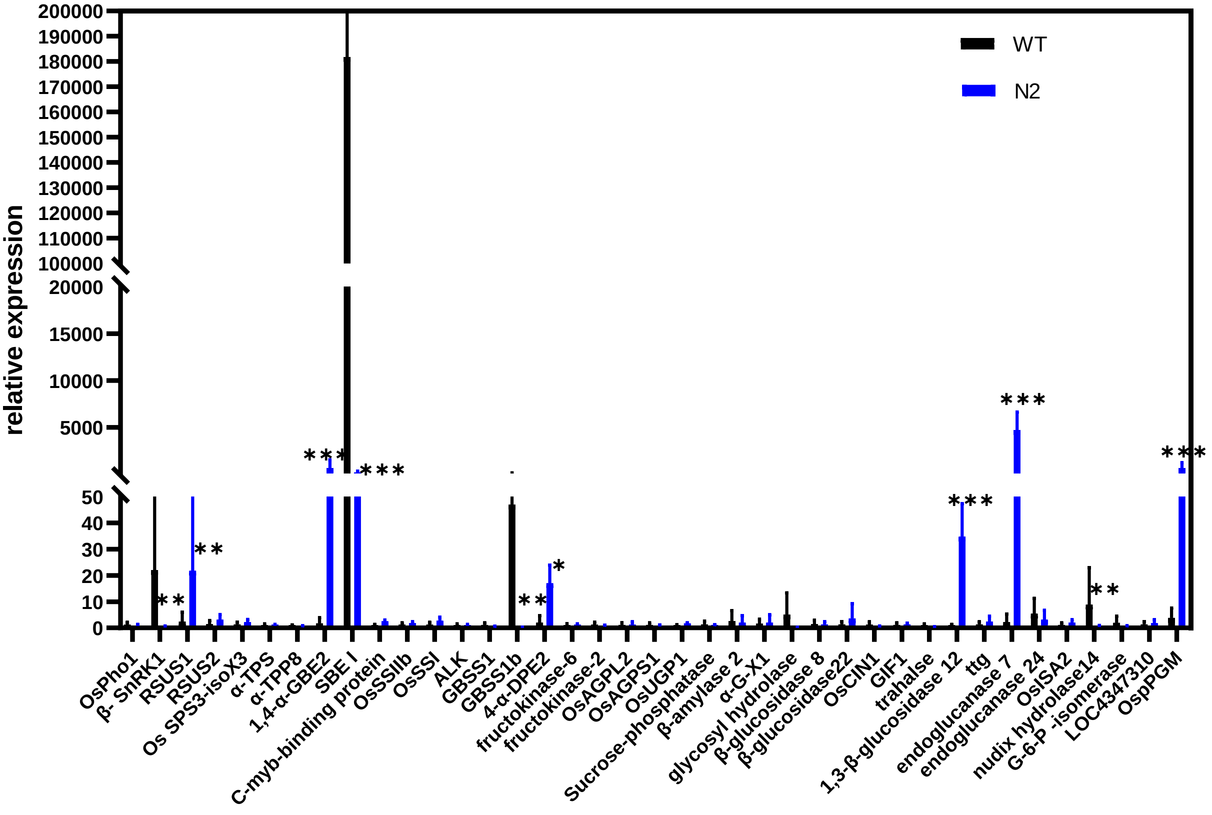


**Fig.S10** The genes of 39 ECs in Sucrose and Starch Metabolism pathway in Mapman of N2 vs WT seeds (3DAP) are verified by qRT-PCR

The qRT-PCR verification in seeds (3DAP) displayed the expressions of OsNAC02 relative genes in its regulatory co-network. All the statistical analyses are performed by student t-test, *, $p < 0.05$, **, $p < 0.01$ and ***, $p < 0.001$.

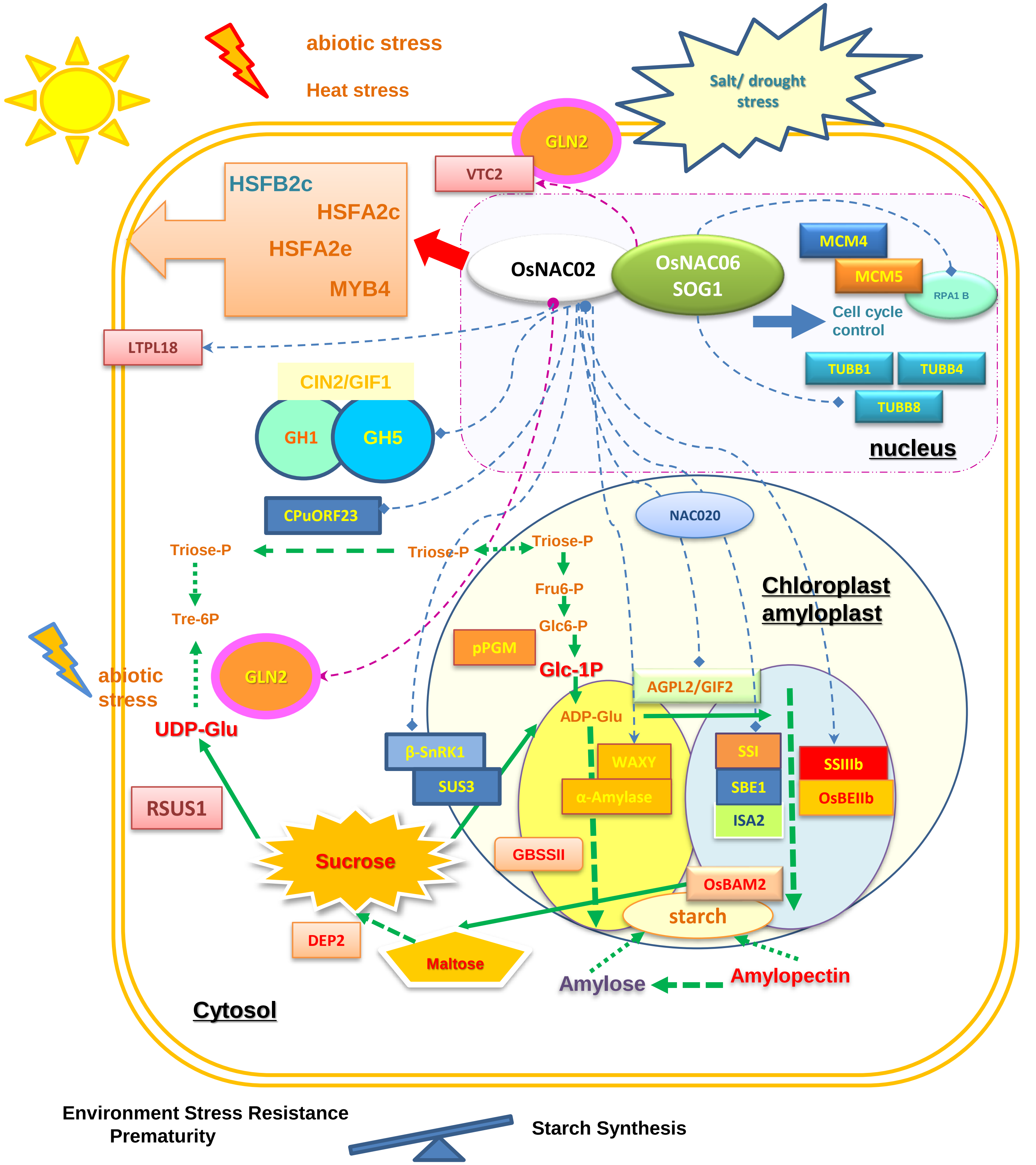


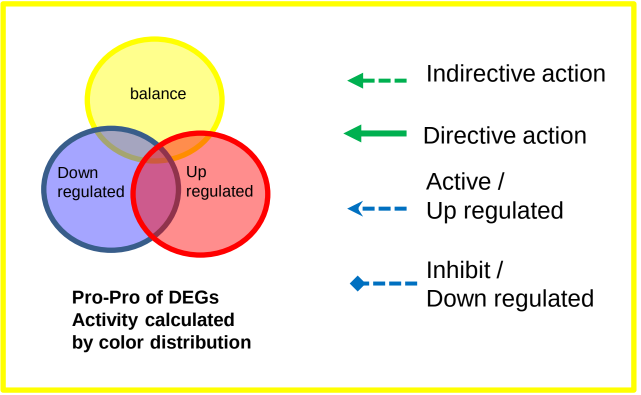


**Fig.S11** The antagonism between energy storage and development requirement under stress in Ko-*Osnac02* mutant

The pro-pro interactions in Ko-*Osnac02* mutant are displayed by arrows with different shapes and different lines.
The expressive activity of more than 30 proteins displayed by genes labeled with circles and rectangles in different colors. The blue is down-regulated while the red is up-regulated.

**Table S5 the genes co-expressed in the down-regulated Venn diagram (N2 vs N3 vs WT , Fig 4b)**

| Co-Venn clusters | Gene ID | Function | Reference |
|---|---|---|---|
| **N2-x\N2-z\N3-1** | LOC_Os10g11889 | The pathogenous stress such as the (*Magnaporthe* L.) induced | (Drogue et al., 2014) |
| **N2-x\N2-2\N3-1** | LOC_Os11g37270 | Ar-AMP have the broad-spectrum potent activity to the multi-drug resistant , which similar to Embryo-specific protein | (Khamis et al., 2015) |
| **N2-2\N2-Z\N3-3** | LOC_Os07g43160 | OsTPP3 as the enzyme in ABA pathways induced by the abiotic stress and expressed high in endosperm | (Yang et al, 2018) |
| **N2-1\N3-3** | LOC_Os04g36750 | α-protein crystallography hsp20 is protein chaperones | (Wang et al., 2014) |
| **N2-1\N2-2** | LOC_Os01g03390 | BBTI7 -Bowman-Birk type bran trypsin inhibitor precursor | (Sonsungsan et al., 2021) |
| **N2-1\N2-2** | LOC_Os08g03410 | Glutelin as storage in endosperm | (Gan et al., 2021) |
| **N3-1\N3-3** | LOC_Os03g63330 | aspartokinase, chloroplast which mainly response to the biotic stress | (Shaar-Moshe et al., 2015) |

**Table S8 the volcano diagram of genes hub in DEGs of the seeds (N2 vs N3 vs WT, Fig 4c)**

| Up-regulated | All DEGs in top 5 | Function |
|---|---|---|
| **1** | LOC_Os02g08150 | CCT/B-box zinc finger protein, putative, expressed |
| **2** | LOC_Os12g08810 | VTC2, putative |
| **3** | LOC_Os01g62810 | regulator of chromosome condensation |
| **4** | LOC_Os04g09430 | cytochrome P45 |
| **5** | LOC_Os09g29210 | OsPUP2 |
| **Down-regulated** | LOC_Os01g01470 | OsNAC20; ONAC020 |
| **2** | LOC_Os02g15070 | glutelin, putative, expressed |
| **3** | LOC_Os11g13980 | hsp20/alpha crystallin family protein, putative, expressed |

**Table S9 the volcano plot of labeled genes in Sucrose and Starch pathway of the seeds (N2 vs N3 vs WT, Fig 4d)**

| Up-regulated | DEGs in the top 5 | Gene name | In Sucrose and Starch pathway |
|---|---|---|---|
| **1** | LOC_Os10g32810 | OsBAM2 | (Kim et al., 2017) |
| **2** | LOC_Os01g12020 | LTPL18 - Protease | (Zhang et al., 2010) |
| **3** | LOC_Os01g71670 | OsGLN2 | (Akiyama et al., 2004) |
| **4** | LOC_Os04g53310 | OsSSIIIb | (Wang et al., 2015a) |
| **5** | LOC_Os07g22930 | GBSSII | (Maung et al., 2021) |
| **Down-regulated** | LOC_Os06g49970 | alpha-amylase | (Damaris et al., 2019) |
| **2** | LOC_Os03g11420 | Os3BGlu6,glycosyl hydrolase family 1 | (Sun et al., 2021) |
| **3** | LOC_Os04g40510 | glycosyl hydrolase family 5 | (Xiang et al., 2019) |
| **4** | LOC_Os03g22790 | beta-amylase | (Wang et al., 2022a) |
| **5** | LOC_Os10g40550 | CPuORF23 | (Shaar-Moshe et al., 2015) |
